# Siderophore interactions drive the ability of *Pseudomonas* spp*.* consortia to protect tomato against ***Ralstonia solanacearum***

**DOI:** 10.1093/hr/uhae186

**Published:** 2024-07-12

**Authors:** Zhengying Shao, Shaohua Gu, Xiaoni Zhang, Jiao Xue, Tao Yan, Saisai Guo, Thomas Pommier, Alexandre Jousset, Tianjie Yang, Yangchun Xu, Qirong Shen, Zhong Wei

**Affiliations:** Jiangsu Provincial Key Lab for Solid Organic Waste Utilization, Jiangsu Collaborative Innovation Center for Solid Organic Waste Resource Utilization, Educational Ministry Engineering Center of Resource-saving fertilizers,National Engineering Research Center for Organic-based Fertilizers, College of Resources and Environmental Sciences, Nanjing Agricultural University, Nanjing 210095, China; Jiangsu Provincial Key Lab for Solid Organic Waste Utilization, Jiangsu Collaborative Innovation Center for Solid Organic Waste Resource Utilization, Educational Ministry Engineering Center of Resource-saving fertilizers,National Engineering Research Center for Organic-based Fertilizers, College of Resources and Environmental Sciences, Nanjing Agricultural University, Nanjing 210095, China; Center for Quantitative Biology and Peking-Tsinghua Center for Life Sciences, Academy for Advanced Interdisciplinary Studies, Peking University, Beijing 100871, China; Jiangsu Provincial Key Lab for Solid Organic Waste Utilization, Jiangsu Collaborative Innovation Center for Solid Organic Waste Resource Utilization, Educational Ministry Engineering Center of Resource-saving fertilizers,National Engineering Research Center for Organic-based Fertilizers, College of Resources and Environmental Sciences, Nanjing Agricultural University, Nanjing 210095, China; Jiangsu Provincial Key Lab for Solid Organic Waste Utilization, Jiangsu Collaborative Innovation Center for Solid Organic Waste Resource Utilization, Educational Ministry Engineering Center of Resource-saving fertilizers,National Engineering Research Center for Organic-based Fertilizers, College of Resources and Environmental Sciences, Nanjing Agricultural University, Nanjing 210095, China; Jiangsu Provincial Key Lab for Solid Organic Waste Utilization, Jiangsu Collaborative Innovation Center for Solid Organic Waste Resource Utilization, Educational Ministry Engineering Center of Resource-saving fertilizers,National Engineering Research Center for Organic-based Fertilizers, College of Resources and Environmental Sciences, Nanjing Agricultural University, Nanjing 210095, China; Jiangsu Provincial Key Lab for Solid Organic Waste Utilization, Jiangsu Collaborative Innovation Center for Solid Organic Waste Resource Utilization, Educational Ministry Engineering Center of Resource-saving fertilizers,National Engineering Research Center for Organic-based Fertilizers, College of Resources and Environmental Sciences, Nanjing Agricultural University, Nanjing 210095, China; Setec Energie Environnement, 97/101 bvd Vivier Merle, Lyon 69003, France; Jiangsu Provincial Key Lab for Solid Organic Waste Utilization, Jiangsu Collaborative Innovation Center for Solid Organic Waste Resource Utilization, Educational Ministry Engineering Center of Resource-saving fertilizers,National Engineering Research Center for Organic-based Fertilizers, College of Resources and Environmental Sciences, Nanjing Agricultural University, Nanjing 210095, China; Jiangsu Provincial Key Lab for Solid Organic Waste Utilization, Jiangsu Collaborative Innovation Center for Solid Organic Waste Resource Utilization, Educational Ministry Engineering Center of Resource-saving fertilizers,National Engineering Research Center for Organic-based Fertilizers, College of Resources and Environmental Sciences, Nanjing Agricultural University, Nanjing 210095, China; Jiangsu Provincial Key Lab for Solid Organic Waste Utilization, Jiangsu Collaborative Innovation Center for Solid Organic Waste Resource Utilization, Educational Ministry Engineering Center of Resource-saving fertilizers,National Engineering Research Center for Organic-based Fertilizers, College of Resources and Environmental Sciences, Nanjing Agricultural University, Nanjing 210095, China; Jiangsu Provincial Key Lab for Solid Organic Waste Utilization, Jiangsu Collaborative Innovation Center for Solid Organic Waste Resource Utilization, Educational Ministry Engineering Center of Resource-saving fertilizers,National Engineering Research Center for Organic-based Fertilizers, College of Resources and Environmental Sciences, Nanjing Agricultural University, Nanjing 210095, China; Jiangsu Provincial Key Lab for Solid Organic Waste Utilization, Jiangsu Collaborative Innovation Center for Solid Organic Waste Resource Utilization, Educational Ministry Engineering Center of Resource-saving fertilizers,National Engineering Research Center for Organic-based Fertilizers, College of Resources and Environmental Sciences, Nanjing Agricultural University, Nanjing 210095, China

## Abstract

The soil-borne bacterial pathogen ***Ralstonia solanacearum*** causes significant losses in Solanaceae crop production worldwide, including tomato, potato, and eggplant. To efficiently prevent outbreaks, it is essential to understand the complex interactions between pathogens and the microbiome. One promising mechanism for enhancing microbiome functionality is siderophore-mediated competition, which is shaped by the low iron availability in the rhizosphere. This study explores the critical role of iron competition in determining microbiome functionality and its potential for designing high-performance microbiome engineering strategies. We investigated the impact of siderophore-mediated interactions on the efficacy of *Pseudomonas spp.* consortia in suppressing ***R. solanacearum***, both *in vitro* and *in vivo*. Our findings show that siderophore production significantly enhances the inhibitory effects of *Pseudomonas* strains on pathogen growth, while other metabolites are less effective under iron-limited conditions. Moreover, siderophores play a crucial role in shaping interactions within the consortia, ultimately determining the level of protection against bacterial wilt disease. This study highlights the key role of siderophores in mediating consortium interactions and their impact on tomato health. Our results also emphasize the limited efficacy of other secondary metabolites in iron-limited environments, underscoring the importance of siderophore-mediated competition in maintaining tomato health and suppressing disease.

## Introduction

The soil-borne pathogen *Ralstonia solanacearum* is a global threat for Solanaceae crop production worldwide. It can infect more than 100 crop species, resulting in substantial yield losses [1, 2]. While this disease is hard to control by conventional means, there is a growing awareness that the root-associated microbiome plays a major role in protecting plants against infection [3–5]. Plant roots are covered with a multispecies biofilm that may directly inhibit pathogens, outcompete them or stimulate the plant immune response [6, 7]. However, despite a multitude of root-associated bacterial strains exhibiting soil-borne plant pathogen suppression under laboratory conditions, designing practical applications with strong efficacy in the field remains challenging [8]. This limitation stems largely from a lack of consideration for the mechanisms driving pathogen control in natural field communities spanning thousands of species. Understanding the mechanisms underlying plant protection is thus critical to the design of sustainable disease control strategies.

Plant-associated microorganisms can prevent disease through a range of mechanisms, including direct pathogen inhibition or resource competition [9]. While direct inhibition is essential for disease control in simplified systems [10–12], resource competition or facilitation becomes the primary driver of pathogen success in a multi-species context such as the natural microbiome [13], starting already with simplified synthetic consortia [14]. Among all nutrients, iron holds a special position, serving as a vital component for microorganisms as an enzyme cofactor in different metabolic activities [15]. Despite being highly abundant in the earth’s crust, iron is a limiting factor for microbial growth due to its extremely low bioavailability. In the rhizosphere, Fe(III) concentrations are usually even lower because aerobic organisms (plants and microorganisms) strongly compete for iron [16]. In response to iron deficiency, many microorganisms synthesize and secrete siderophores, which are low-molecular-weight, high-affinity Fe(III) chelators, to mobilize iron from iron-bearing minerals or organic matter complexes [15, 17, 18].

Harnessing siderophores to make iron unavailable to pathogens offers a potent mechanism for controlling soil-borne diseases [16, 17]. For instance, certain *Pseudomonas* species have demonstrated an ability to suppress pathogenic bacteria through the secretion of siderophores, effectively cutting off iron supply to pathogens [19, 20]. *Pseudomonas aeruginosa* FP6, a biocontrol strain inhibiting the fungal pathogen *Rhizoctonia solani*, lost almost all its inhibitory activity against *R. solani* after FeCl_3_ supplementation, highlighting the role of iron competition as the main mechanism for pathogen control [20]. Extensive literature documents the variety and functionality of siderophores produced by *Pseudomonas* spp. [21, 22], as well as their contributions to disease prevention [23]. Pyoverdines, a diverse class of non-ribosomal peptides that possess the most complex chemical structures among *Pseudomonas* siderophores, have been extensively studied, and over 50 structurally distinct pyoverdines have been identified [24]. The structural complexity of siderophores is noteworthy for its diversity, exhibiting remarkable variation even within a single bacterial strain [24]. Here we focused on *Pseudomonas* spp. siderophores due to their great structural diversity and substantial potential for biological control applications [25, 26]. Furthermore, pyoverdines are highly specific. Their uptake requires very specific receptors, driving the same molecule to promote or inhibit bacterial strains depending on their ability to use it [27]. Thus, siderophore-mediated competition for iron shapes ecological interactions between microorganisms [28]. In the multispecies rhizosphere microbiome, these interactions may ultimately affect the performance and health of plant hosts [29, 30].

Although many studies have approached the importance of iron as the driver of pathogen control, most conclusions are drawn from pairwise interactions between monocultures [31, 32]. This finding may not reflect the reality of a multispecies microbiome, in which thousands of species simultaneously interact. In previous studies, we employed bacterial strains that exclusively engage in nutritional competition with the tomato pathogen to probe the dynamics between the invasive pathogen *R. solanacearum* and bacterial consortia mediated by bacterial siderophores [33]. However, several bacteria both compete for resources and produce bioactive secondary metabolites, raising the question of which mechanisms are more relevant to control diseases in the rhizosphere [34]. Further, amidst these discussions on antibiotic compounds, the precise role of microbial siderophores in combating pathogen encroachment remains somewhat ambiguous and often limited to strain-specific case studies with limited potential for extrapolation.

In this study we used tomato as model plant, and selected seven *Pseudomonas* spp*.* strains that were previously reported to produce both siderophores and a range of secondary metabolites, including the broad-spectrum polyketide 2,4-diacetylphloroglucinol (DAPG). We focused on DAPG as a reference metabolite as it is well described to inhibit the growth of the pathogen *R. solanacearum* and prevent bacterial wilt in tomato plants [35]. Using these strains, we assembled a total of 49 consortia of *Pseudomonas* spp*.* genotypes covering various biodiversity levels. We grew these strains under iron-limiting conditions, which matches realistic iron bioavailability in the rhizosphere, and iron-rich conditions providing a baseline without the effect of siderophores. We determined the relative effects of siderophores and other metabolites on pathogen growth, invasiveness and pathogenicity. This suppressive activity of the *Pseudomonas* spp. consortia against tomato wilt disease caused by *R. solanacearum* was validated in a greenhouse assay. In this article we mainly focus on the direct effects of siderophores and their mediating interactions on the ability of bacterial communities to resist tomato pathogen invasion by using antagonistic bacteria.

## Results

### Siderophores affected the inhibition of pathogen growth in consortium members

**Figure 1:**
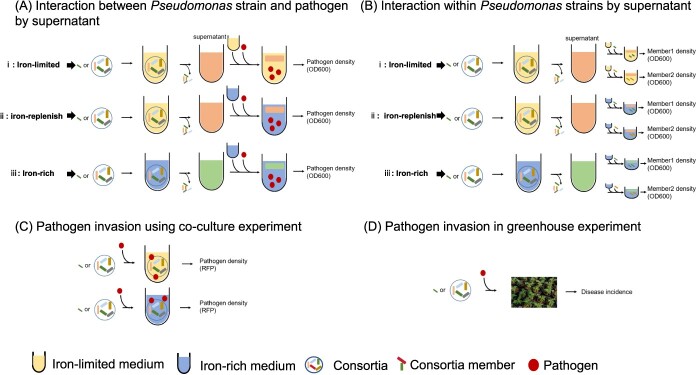
Schematic figure depicting the experimental design. **A**, **B** Determining the interaction between bacterial consortia and pathogen *R. solanacearum*, and within consortia by a supernatant experiment. We set up three treatments (iron-rich, iron-limited, and iron-replenish) in the supernatant experiments. The iron-rich condition leads to production of various secondary metabolites, but no siderophores. The iron-limited condition leads to production of secondary metabolites including siderophores. The iron-replenish condition forms an internal control in which the effect of siderophores has been cancelled by iron supplementation, while keeping other metabolites untouched. The siderophore-mediated effect was calculated by subtracting the impact of supernatant from iron-replenished to iron-limited conditions on pathogen growth *in vitro*. **C** Pathogen inhibition rate of bacterial consortia against pathogen *R. solanacearum* was determined using co-culture experiments. **D** In a final step, the impact of siderophore-mediated interactions on the resistance of tomato plants against *Ralstonia* wilt was determined in a greenhouse experiment with tomato plants inoculated with different *Pseudomonas* spp*.* consortia and the pathogen *R. solanacearum.*

**Figure 2:**
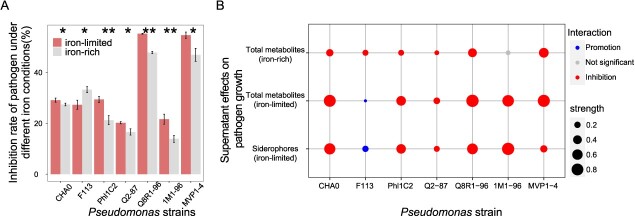
Inhibition rate of seven *Pseudomonas* strains against pathogen *R. solanacearum* QL-Rs1115 under iron-rich and iron-limited co-culture conditions (**A**) and different metabolite-mediated effects of *Pseudomonas* strains on pathogen growth under iron-limited or iron-rich conditions (**B**). Different metabolites represent distinct cell-free supernatants under different iron conditions. Listed from top to bottom in panel **B**: total metabolites harvested under iron-rich conditions; total metabolites obtained under iron-limited conditions; and siderophores collected under iron-limited conditions. The size of the bubbles represents the strength of the facilitative or inhibitive relationships, respectively. Student’s *t*-test was used with a defined siderophore-mediated effect falling between 0.15 and −0.15 considered non-significant in panel **B**.

To determine the growth inhibition of the tomato pathogen by consortium members, co-culture experiments involving both *Pseudomonas* strains and the pathogen were performed under both iron-limited and iron-rich conditions, where metabolite-mediated effects and direct nutritional competition were involved (Fig. 1C). Under both iron-rich and iron-limited environments, all seven tested antagonistic *Pseudomonas* strains suppressed the growth of tomato pathogen *R. solanacearum* (QL-Rs1115), albeit at varying rates of inhibition. However, the inhibition rates of the *Pseudomonas* strains were influenced by iron availability (two-tailed *t*-test, *t* = 3.87, df = 20, *P* < 0.01), with most *Pseudomonas* strains displaying a higher inhibition rate in iron-limited conditions compared with iron-rich environments. Specifically, strains CHA0, Phl1C2, Q2-87, Q8R1-96, 1 M1-96, and MVP1-4 exhibited significantly higher pathogen inhibition rates under iron-limited conditions compared with iron-rich conditions, whereas strain F113 displayed an enhanced pathogen inhibition rate under iron-rich conditions (Fig. 2A).

Iron deficiency led to both an increase in siderophore production and a reduction in *Pseudomonas* growth (Supplementary Data Fig. S1), in line with findings from prior studies [13, 36]. To specifically explore the metabolite-mediated effects of *Pseudomonas* strains on pathogen growth, their supernatants under iron-rich and iron-limited conditions were collected (Fig. 1A). Distinct from co-cultivation assays (Fig. 1C), these supernatant experiments (Fig. 1A) focused exclusively on the impacts mediated by metabolites, eliminating any interference from nutritional competition. The results showed that different iron concentrations significantly influenced the metabolite-mediated effects of all tested *Pseudomonas* strains on pathogen growth (Fig. 2B and Supplementary Data Fig. S2). Most supernatants derived from *Pseudomonas* strains demonstrated an inhibitory effect on *R. solanacearum* growth under both iron-rich and iron-limited conditions, the inhibition being more pronounced under iron scarcity, except for strain F113 (Fig. 2B and Supplementary Data Fig. S2). To uncover which fraction of this effect is mediated by siderophores, we created an iron-replenish treatment by adding enough FeCl_3_ to deactivate the siderophores in the supernatant, while keeping potential inhibitory effects mediated by other metabolites. By subtracting the interactions measured in iron-replenished conditions, we were able to specifically identify the effects mediated by siderophores. We found that siderophores produced by seven *Pseudomonas* spp. strains showed distinct effects on pathogen growth: siderophores produced by strain F113 promoted pathogen growth, while *Pseudomonas* spp. strains CHA0, Q2-87, Q8R1-96, MVP1-4, Phl1C2, and 1M1-96 produced siderophores that inhibited pathogen growth, strain 1M1-96 showing the strongest inhibitory effect (Fig. 2B). Siderophore-mediated antagonistic effects of antagonistic bacteria were stronger than other metabolite-mediated effects under iron-limited conditions, strain F113 being the sole exception (Fig. 2B and Supplementary Data Fig. S2).

**Figure 3:**
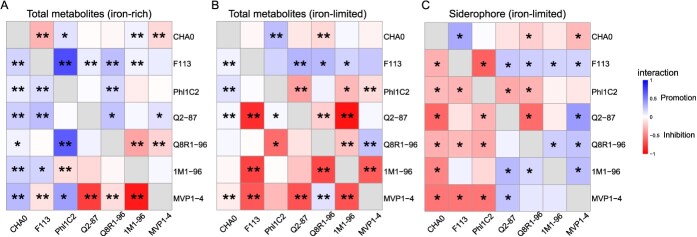
Interactions between *Pseudomonas* strains under different iron conditions. Different metabolites represent different cell-free supernatants under different iron conditions. **A**, **B** Total metabolite-mediated effects between *Pseudomonas* strains under iron-rich (**A**) and iron-limited (**B**) conditions. **C** Siderophore-mediated effects between *Pseudomonas* strains under iron-limited conditions. The color palette represents the strength of the facilitative or inhibitory relationships. Asterisks (*) denote the presence of significant inhibitory or facilitative effects (Student’s *t*-test, ^*^: *P* < 0.05, ^**^P < 0.01, but a siderophore-mediated effect between 0.15 and −0.15 is considered non-significant).

### Iron deficiency affected other metabolite-mediated effects of consortium members on pathogen growth

Siderophore production is part of the stress response of bacteria facing iron-limited environments. Consequently, in iron-rich settings, strains typically exhibit low levels of siderophore production [13]. Our results revealed that *Pseudomonas* strains exhibit minimal to negligible siderophore production when cultivated in iron-rich medium (50 μM Fe(III); Supplementary Data Fig. S1B), suggesting that the effects observed under iron-rich conditions were primarily influenced by other metabolites. Meanwhile, in the iron-replenish treatment, the addition of adequate Fe(III) resulted in the neutralization of the siderophore, emphasizing the role of other metabolites. To further understand the impact of iron availability on other metabolites, we compared the effects of other metabolites on *R. solanacearum* growth under iron-rich and iron-limited conditions. Our findings demonstrate that other metabolite-mediated effects under iron-limited conditions (iron concentration ~6 μM) [37] differed significantly from those under iron-rich conditions in all *Pseudomonas* strains (Supplementary Data Fig. S2). Specifically, the inhibitory effects mediated by other metabolites were significantly reduced under iron-limited conditions (two-tailed *t*-test, *t* = 5.02, df = 20, *P* < 0.01). Moreover, other metabolites of the seven *Pseudomonas* strains mainly mediated inhibitory effects on pathogen growth under iron-rich conditions (Supplementary Data Fig. S2).

*Pseudomonas* spp. directly inhibits the growth of tomato pathogen *R. solanacearum* through the production of DAPG, which is a broad-spectrum antagonistic secondary metabolite to the pathogenic bacteria [38]. Phloroglucinol (PG), serving as a crucial precursor of DAPG, plays a crucial role in the direct inhibition mediated by *Pseudomonas* strains. In addition to DAPG, we observed a decrease in the concentration of its precursor molecule, PG, under iron limitation. This observation is consistent with previous reports indicating that DAPG production can be enhanced by shifting the balance between precursors and the final molecule [39]. In our study, we observed that the PG content of *Pseudomonas* strains differed under different iron conditions, with specific strains (CHA0, Phl1C2, Q2-87, 1M1-96, and MVP1-4) showing a significant decrease in PG content under iron limitation (Supplementary Data Fig. S3A). Further, we examined the expression of *PhlD*, a gene responsible for the last step of DAPG biosynthesis, in seven strains. Only two strains (Q8R1-96 and 1M1-96) displayed significant downregulation in iron-limited conditions, while the others showed no notable difference (Supplementary Data Fig. S3B). These results indicate that iron-limited conditions may impact secondary metabolite production independently of gene expression, potentially by altering post-transcriptional regulation [40].

### Siderophore production altered pairwise interactions between *Pseudomonas* strains

A total of 42 pairwise interactions between *Pseudomonas* strains were investigated through supernatant experiments (Fig. 1B). Similarly, the siderophore-mediated pairwise interaction relationships were confirmed by removing the effects of other metabolites from total metabolites in iron-limited conditions (iron concentration ~6 μM; Fig. 1B). Iron limitation affected pairwise interactions between *Pseudomonas* strains (Fig. 1B and Supplementary Data Fig. 3). Under the iron-rich condition (iron concentration = 50 μM), other metabolite-mediated effects between *Pseudomonas* strains were predominantly facilitative, with 20 out of 42 pairwise interactions significantly promoting growth (accounting for 47.62%). In contrast, significant competition was less common, with 21.43% (9 out of 42 pairwise interactions) showing growth inhibition, while the remaining differences were not significant (Fig. 3A). Under iron-limited conditions, the total metabolite-mediated promotion between *Pseudomonas* strains account for 23.81% (10 out of 42 pairwise interactions significantly promoted growth; Fig. 3B). After further differentiating the effects of siderophores and other metabolites, we found that siderophores mainly mediated inhibition between strains (18 out of 42 pairwise interactions significantly inhibited growth, 42.86%, 12 out of 42 pairwise interactions significantly promoted growth, 28.57%; Fig. 3C). Iron restriction intensified metabolite-mediated inhibition between *Pseudomonas* strains, with siderophores emerging as the primary mediator of inhibition among the strains. Further comparison of the pairwise interactions with genomics predictions based on siderophore–receptor pairs revealed a high consistency rate of 90% [27].

### Siderophore-mediated interactions within consortia determined the disease resistance of tomato plants inoculated with consortia

To evaluate the influence of iron concentration on the microbial communities' ability to suppress pathogen invasion of tomato plants, we built 42 multi-strain bacterial consortia by combining seven *Pseudomonas* strains and conducted co-culture experiments with pathogen *R. solanacearum* (Fig. 1C and Supplementary Data Table S2). In this co-cultivation system, a lower pathogen abundance indicated a higher efficacy of the bacterial consortia in suppressing pathogen invasion. Co-culture experiments showed that the different consortia significantly inhibited the growth of the plant-pathogenic bacterium *R. solanacearum* QL-Rs1115 under both iron-limited and iron-rich co-culture conditions, with notable differences in inhibition rates between the two conditions. Iron restriction intensified the inhibitory effect of most consortia on pathogen growth, with 28.58% of the consortia significantly enhancing the suppression of pathogen *R. solanacearum*, while 11.90% of the consortia significantly weakened it (Fig. 1C and Supplementary Data Fig. S4). There was no significant correlation between the number of strains in the consortium and pathogen abundance when the consortium was co-cultured with the pathogen *R. solanacearum* under iron-limited conditions (Supplementary Data Fig. S5A). However, we observed a significant correlation between siderophore production and pathogen abundance in co-culture conditions (*R*^2^ = 0.12, *F*_1,40_ = 5.54, *P* < 0.05; Supplementary Data Fig. S5B).

**Figure 4:**
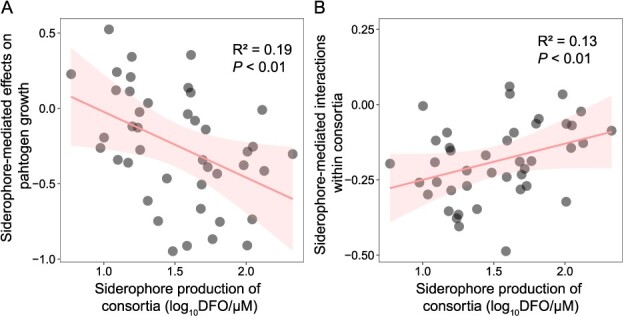
Siderophore-mediated effects on pathogen growth and siderophore-mediated interactions within consortia correlated with the siderophore production. **A** Interactions between siderophore production of *Pseudomonas* consortia and siderophore-mediated effects on *Ralstonia* growth in the supernatant experiments. **B** Interactions between siderophore production of *Pseudomonas* consortia and the siderophore-mediated interaction (quantified as mean siderophore-mediated growth effect by multiple strain consortia on each consortium member) within the consortia in the supernatant experiments.

To investigate the specific role of siderophores in the disease suppressiveness of bacterial consortia, the effects of siderophores and other metabolites on pathogen suppression were assessed through supernatant experiments (Fig. 1A). Under iron-rich conditions (iron concentration = 50 μM), the growth of the pathogen *R. solanacearum* was primarily inhibited by the total metabolites of the consortia, with 61.90% of consortia showing pathogen growth inhibition (Supplementary Data Fig. S6). However, under iron limitation (iron concentration ~6 μM), pathogen inhibition by total metabolites was intensified, with 95.24% of the consortia exhibiting pathogen growth suppression (Supplementary Data Fig. S6). Iron limitation also significantly restricted the growth of all the *Pseudomonas* consortia and increased the production of siderophores (Supplementary Data Fig. S7A, *t* = 11.74, df = 148.08, *P* < 0.01; Supplementary Data Fig. S7B, *t* = −13.89, df = 265.97, *P* < 0.01). The siderophore production of the community exhibited a significant and positive correlation with biomass, underscoring the contribution of siderophores to community growth under iron-limited conditions (*R*^2^ = 0.55, *F*_1,47_ = 57.37, *P* < 0.01; Supplementary Data Fig. S7C). To clarify the role of siderophores in metabolite-mediated inhibition of multi-strain bacterial consortia (42 *Pseudomonas* consortia), we distinguished siderophore- and other metabolite-mediated effects from total metabolites under iron-limited conditions by the method described above. Siderophores predominantly mediated the inhibitory effect on pathogen growth (73.81% of all consortia inhibited pathogen growth), while 66.67% of all consortia relied on other metabolites for pathogen growth inhibition (Supplementary Data Fig. S6). The direct effects of *Pseudomonas* consortia mediated by siderophores were significantly correlated with siderophore production (*R*^2^ = 0.19, *F*_1,40_ = 9.32, *P* < 0.01): the higher the siderophore production for the consortium, the stronger the suppression of pathogenic bacteria (Fig. 4A). The relatively low correlation coefficient may be attributed to the dual promoting and inhibitory effects of siderophores. The interaction relationship mediated by siderophores holds greater significance compared with the yield of siderophores. However, there was no correlation between pathogen abundance and siderophore-mediated effects on tomato pathogen growth, indicating that, at the whole-consortium level, siderophore-mediated effects had little effect on pathogen suppression (Supplementary Data Fig. S8A). Similar results were observed for other metabolites (Supplementary Data Fig. S8B).

**Figure 5:**
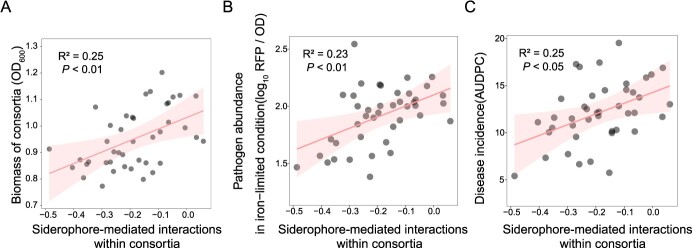
Siderophore-mediated interactions within consortia predicts the ability to suppress pathogen invasion and biomass of consortia. **A** Siderophore-mediated interactions explain consortium biomass under iron-limited conditions. **B** Co-culture experiments between the pathogen and individual bacterial consortium show that pathogen abundance correlated with siderophore-mediated interactions within consortia in the supernatant experiment under iron-limited conditions. Pathogen abundance is indicated by pathogen density relative to total bacterial density [log_10_ (RFP/OD_600_)]. **C** Area under the disease progress curve (AUDPC) of tomato plants inoculated with consortia correlated with siderophore-mediated interactions within consortia in the supernatant experiments.

Together, these findings indicate that iron deficiency induces alterations in the pairwise interactions among members of *Pseudomonas* consortia and between *Pseudomonas* spp. and *R. solanacearum.* We hypothesize that iron concentration may affect the internal interactions within the microbial community, thereby altering its ability to resist pathogen invasion. Initially, we measured and quantified the interactions occurring within the microbial community by assessing the effects of the community’s metabolites on the growth of each member, which we term ‘interaction strength’ (Fig. 1B). All the parameters related to siderophore-mediated interactions within consortia were from 42 consortia, single-species consortia excluded. Our findings demonstrate a positive correlation between siderophore yield and interactions within the consortia (*R*^2^ = 0.13, *F*_1,40_ = 5.77, *P* < 0.05; Fig. 4B), possibly attributable to the promotion of growth in *Pseudomonas* strains through facilitative siderophore production. Furthermore, we observed significant strain identity effects on siderophore production and siderophore-mediated interaction strength, while the strain richness effect was non-significant (*P* > 0.05; Supplementary Data Tables S3 and S4). Specifically, the presence of strain CHA0 positively impacted both siderophore production and siderophore-mediated interaction strength within consortia (Supplementary Data Tables S3 and S4). It is worth noting that there was a significant positive correlation between interactions within consortia mediated by siderophore and microbial community biomass (*R*^2^ = 0.25, *F*_1,40_ = 13.44, *P* < 0.01; Fig. 5A), highlighting the direct impact of interaction within consortia on the growth of microbial communities. Furthermore, in cocultures under iron-limited conditions, pathogen inhabitation was best predicted by siderophore-mediated interaction strength within consortia (*R*^2^ = 0.23, *F*_1,40_ = 12.24, *P* < 0.01; Fig. 5B). The more intense the competition for iron within consortia mediated by siderophores, the lower the relative abundance of the pathogen. Siderophore-mediated interactions emerged as the key determinant of pathogen suppression, superseding the direct effect of other metabolites. Through a general linear modeling approach, we compared the impacts of siderophore-mediated interactions within consortia, siderophore-mediated effects on pathogen growth, siderophore production, other metabolite-mediated interactions within consortia, other metabolite effects, and consortia biomass on pathogen inhibition. Our results highlight that, among these factors, siderophore-mediated interactions were identified as the most critical element influencing the ability of bacterial consortia to suppress pathogen invasion (Table 1).

**Table 1:** General linear mixed model comparing contributions of siderophore-mediated interactions within consortia, siderophore-mediated effects, siderophore production, other metabolite-mediated interactions within consortia, other metabolite-mediated effects, and consortia biomass on pathogen inhibition by inoculated consortia.

	Pathogen abundance			Disease incidence (AUDPC)		
Variable	Df	F	P	Df	F	P
Interactions_sid^a^	↑1	38.07	< 0.001	↑1	27.49	< 0.001
Biomass	↑1	23.77	< 0.001	↑1	16.21	< 0.001
Siderophore production	↓1	3.41	0.07	↓1	7.10	0.009
Interactions_other^b^	↓1	0.58	0.45	↑1	0.02	0.89
Effect_sid^c^	↑1	0.19	0.66	↑1	0.37	0.55
Effect_other^d^	↑1	4.11	0.05	↑1	0.005	0.94
Residuals	119			119		
Model summary	AIC, 314.20; *R*^2^ = 0.37			AIC, 327.49; *R*^2^ = 0.30		

^a^Siderophore-mediated interactions within consortia in the supernatant experiments. ^b^Other metabolite-mediated interactions in the supernatant experiments. ^c^Siderophore-mediated effects of consortia on pathogen growth in the supernatant experiments. ^d^Other metabolite-mediated effects in the supernatant experiments. Significant effects (*P* < 0.05) are highlighted in bold and the up and down arrows denote positive and negative effects, respectively.^ e^Degree of freedom.

**Figure 6:**
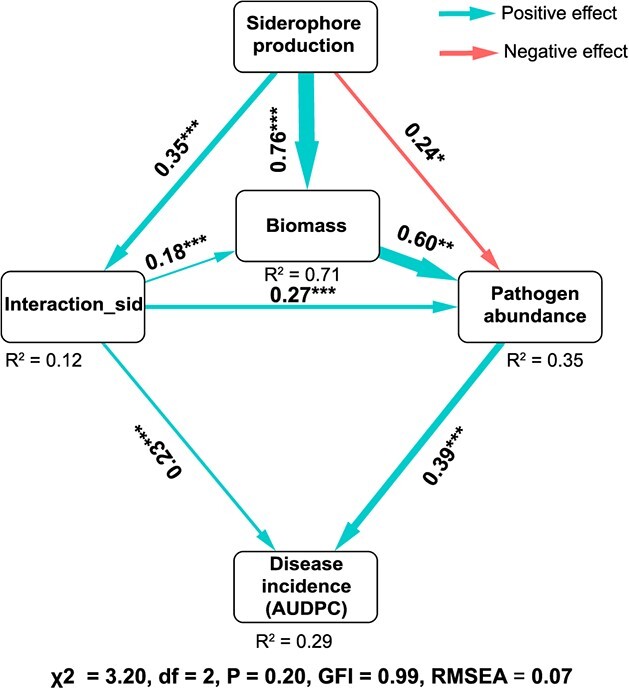
Structural equation model linking siderophore production, siderophore-mediated interactions, and biomass with pathogen abundance and disease incidence (AUDPC) of plant bacterial wilt. Siderophore production, siderophore-mediated interactions in supernatant experiments, and biomass were major contributors to pathogen abundance in co-culture experiments. In the rhizosphere invasion experiment, siderophore-mediated interactions were crucial for the spread of bacterial wilt disease. *R*^2^ denotes the proportion of variance explained. The numbers on the arrows denote indicate the effect size of the relationship. The width of the arrows is proportional to the strength of path coefficients. Asterisks (*) indicate significant correlation (^*^P < 0.1, ^**^P < 0.05, ^***^P < 0.01).

We finally conducted a greenhouse experiment to verify the correlation between the disease resistance of tomato plants and siderophore production associated with different consortia in a natural environment (Fig. 1D). Disease incidence of tomato plants gradually increased over time following pathogen *R. solanacearum* inoculation (Supplementary Data Fig. S9A). Inoculation with most *Pseudomonas* consortia effectively decreased bacterial wilt disease incidence in tomato plants. By the end of the disease period, the average disease incidence of tomato plants with *R. solanacearum* alone was 66.67%, while tomato seedlings treated with both consortia and *R. solanacearum* exhibited a lower disease incidence rate of 57.65% (Supplementary Data Fig. S9A). The greenhouse results showed that there was a significant difference in the area under the disease progress curve (AUDPC) of tomato seedlings inoculated with and without microbial communities (*t*-test, *t* = −6.16, df = 2.54, *P* < 0.05; Supplementary Data Fig. S9B). However, the richness and yield of siderophores cannot well explain the tomato disease severity (Supplementary Data Fig. S10). Similarly, the direct effects of metabolites, whether mediated by siderophores or other metabolites, did not exhibit a significant correlation with plant disease severity (Supplementary Data Fig. S11A and B). Consistent with the co-culture results, siderophore-mediated interaction strength within consortia, as well as biomass of the consortia, correlated with disease suppressiveness (AUDPC) of inoculated consortia, especially for the siderophore-mediated interaction strength within consortia (Table 1). Stronger competition mediated by siderophores within the consortium corresponded to an increased ability of the consortium to suppress pathogen invasion in the tomato rhizosphere (*R*^2^ = 0.25, *F*_1,40_ = 13.65, *P* < 0.01; Fig. 5C). For other metabolites, the interactions within consortia did not significantly influence pathogen invasion within the consortia (Supplementary Data Fig. S11C).

We constructed a structural equation model (SEM) to describe the relationships among siderophore production, the biomass of the consortia, the strength of the interaction within consortia, pathogen abundance in co-culture experiments, and the disease suppressiveness of the consortia (Fig. 6). Our results showed that both biomass and siderophore-mediated interactions exerted direct positive effects on pathogen inhibition rates, as measured by relative pathogen abundance, with biomass and siderophore-mediated interactions showing effects of 0.60 (*P* = 0.00) and 0.27 (*P* = 0.00), respectively. Additionally, siderophore production also directly affected the pathogen abundance (direct effect −0.24, *P* = 0.063; Fig. 6 and Supplementary Data Table S5). The abundance of pathogens and siderophore-mediated interactions both exerted significant positive influences on disease incidence. The direct effects of pathogen abundance and siderophore-mediated interactions on disease incidence were 0.39 (*P* = 0.00) and 0.23 (*P* = 0.01), respectively (Supplementary Data Table S5). Furthermore, siderophore production exhibited an indirect effect on disease incidence (indirect effect 0.21, *P* = 0.00; Supplementary Data Table S5). Under iron-limited conditions, the siderophores produced by bacterial consortia played a crucial role in countering pathogen invasion, achieved by either influencing the growth or mediating the interactions within consortia.

### Effects of siderophores on other metabolite-mediated effects in bacterial consortia

The supernatant experiment showed that iron deficiency leads to alterations in other metabolite-mediated effects on pathogen growth (Supplementary Data Fig. S6). In both iron-rich and iron-limited conditions, other metabolites primarily mediate the inhibitory effects of microbial communities on pathogen growth. More specifically, under iron-rich conditions 61.90% of microbial communities exhibit inhibition, while this percentage increases to 66.67% under iron-limited conditions. Notably, a significant difference was observed between these two conditions (Supplementary Data Fig. S6). Additionally, we identified a positive correlation between the change in the effect of other metabolites on pathogen growth and siderophore production under iron-limited conditions (*R*^2^ = 0.25, *F*_1,40_ = 13.41, *P* < 0.01; Supplementary Data Fig. S12). This correlation suggests that an increase in siderophore production may lead to a reduction in the inhibitory effect mediated by other metabolites on pathogens.

## Discussion

Natural products play a crucial role in microbial interactions, influencing both growth and the functioning of bacterial communities [41, 42]. Siderophores, being among the most critical metabolites in iron-deficient rhizospheres, are often recognized as key biocontrol agents for their ability to directly inhibit pathogen growth due to their high affinity for iron. Bacteria produce a great diversity of siderophores, with different species and even the same species generating various types, leading to intricate interactions among microorganisms [43, 44]. However, the role of siderophores in mediating the interactions between beneficial strains and their effects on other metabolites is commonly ignored, potentially leading to inaccurate evaluation of the application of certain biocontrol agents for suppressing plant disease. Consequently, our study focused on investigating the influence of siderophore-mediated interactions on tomato pathogen suppression by using the artificially synthesized *Pseudomonas* consortia capable of producing siderophores, along with the antagonistic substances like DAPG, which exert inhibitory effects on the tomato pathogenic bacterium *R. solanacearum* under iron-rich conditions. The effects mediated by other metabolites on pathogen growth were also studied. Our findings revealed that: (i) the production of siderophores intensified the suppression of pathogens by *Pseudomonas* strains or consortia in the majority of cases; (ii) siderophore-mediated interactions within *Pseudomonas* consortia determined the suppression of the pathogen by bacterial community; and (iii) iron deficiency results in changes in the effects of other metabolites on pathogen growth, potentially linked to the production of siderophores. These findings highlight the importance of siderophore-mediated interactions and siderophore production in suppressing bacterial wilt of tomato plants.

### Effects of siderophores produced by *Pseudomonas* strains on pathogen growth

Different siderophores may exhibit different interaction relationships due to siderophores and their receptor structures [45]. We found that different *Pseudomonas* siderophores exhibited different effects on the pathogenic bacterium *R. solanacearum*, both *in vitro* and in tomato, with most of them displaying inhibitory effects on the pathogen *R. solanacearum*. Extensive research has illustrated the pivotal role of siderophores produced by biocontrol agents in the suppression of pathogenic bacteria, the level of suppression positively correlating with increased siderophore production [31, 46]. Consistent with these findings, we observed that the production of competitive siderophores by *Pseudomonas* strains intensified the inhibitory effect on the pathogen under iron-limited conditions. Likewise, the inhibition of the pathogen by the *Pseudomonas* consortia was enhanced under iron-limited conditions. Siderophore-mediated effects of *Pseudomonas* consortia on tomato pathogen growth were mainly inhibited and significantly correlated with siderophore production. This may be attributed to the competitive siderophore produced by *Pseudomonas* spp. Nutrient deficiencies often lead to the regulation of metabolism in bacteria. For example, *Candida maltosa* increased the secretion of intermediates of aromatic amino acid catabolism due to carbon and nitrogen starvation in yeast cells [47]. Similarly, the *zinT* gene, encoding the ZinT protein with its unique high-affinity Zn^2+^ binding site, was upregulated by >2-fold (*P* < 0.05) in *Escherichia coli* cells cultured under Zn^2+^-depleted conditions [48]. Siderophore synthesis serves as a stress response to iron limitation, contributing to the enhanced inhibitory effect on pathogen growth. Nonetheless, within antagonistic *Pseudomonas* consortia, the quantity of siderophore production does not sufficiently account for the degree of pathogen suppression observed. This discrepancy may be due to the diverse structures of siderophores, which might impart varying effects. Furthermore, the direct effects on pathogens mediated by siderophores, in conjunction with other metabolites, did not correlate straightforwardly with the observed suppression of pathogens. This suggests that other, more complex interactions within the consortia may play significant roles in pathogen inhibition of tomato plants.

### Effects of siderophore-mediated interactions within consortia on the disease resistance of tomato plants

The efficacy of probiotics in inhibiting the growth of pathogenic bacteria has been well documented, yet the reliance on single probiotics presents challenges such as inconsistent effects and a limited spectrum of biological control against pathogens [49]. Research has suggested that employing a multi-bacterial approach could enhance the effectiveness of biocontrol exhibited by single strains [50, 51]^.^ Despite this, the interactions among different microorganisms are often overlooked. In this study, we found that siderophore production significantly altered the pairwise interactions between members of the bacterial consortia growing *in vitro* or on tomato roots. The siderophore-mediated pairwise interactions in our results were highly consistent with the genome prediction [27], indicating the reliability of the siderophore-mediated effects and methods used in this paper. Therefore, we further investigated the role of siderophore-mediated interactions within consortia in the suppression of pathogens. Our findings suggest that the growth of consortia was driven by siderophore-mediated interactions within consortia (Fig. 5A). More importantly, it was found that siderophore-mediated interactions could better explain pathogen growth inhibition (Fig. 5B). The metabolite-mediated interaction strength within consortia proved more effective in predicting disease suppressiveness than the direct effect of metabolites when bacterial communities were combating pathogenic bacteria. This finding aligns with previous research demonstrating that siderophore-mediated interactions significantly influence the disease-suppressive capacity of bacterial consortia within iron-limited rhizosphere environments [33]. Such insights are supported by additional studies highlighting the critical role of community interactions in enhancing the functionality of consortia [30, 52]. Notably, the intensity of interactions, particularly competitive ones, plays a significant role in suppressing pathogens [30]. Our investigations reveal that heightened competition over siderophores within a consortium markedly increases its capability to suppress pathogen invasions of tomato plants. This bolstered resistance may be due to the diminished probability of invading pathogens accessing siderophores, effectively limiting their growth and spread.

Iron competition is only one of the many factors affecting bacterial interactions in the rhizosphere. The high predictive power of siderophores indicates that iron limitation may be even more relevant than competition for other resources, such as organic compounds [53–55]. Among these factors, iron's availability stands out as a critical regulator of bacterial growth and interactions due to its scarcity in the rhizosphere, which constrains the proliferation of all bacteria [28]. Our prior research, examining over 2000 strains, revealed that siderophore production is prevalent among tomato rhizosphere bacteria and significantly impacts pathogen growth [13]. Moreover, another study showed that effects mediated by bacterial siderophores played a non-negligible role in bacterial interactions and pathogen growth [33]. Both studies indicate that siderophores explain 10–40% (*R*^2^ ranges from 0.10 to 0.42) of the effects mediated by total metabolites on the interactions within the bacterial consortia and pathogen growth. Additionally, while bacteria secrete a myriad of metabolites and other secondary metabolites crucially contribute to bacterial interactions, the impact of siderophores cannot be underestimated. It has been reported that pyoverdines, produced by *Pseudomonas* strains, possess the capability to inhibit phytopathogen growth [56]. Nonetheless, the mechanisms through which pyoverdines or other siderophores mediate interactions within bacterial communities remain to be fully understood. Given the diversity of siderophore structures produced by the tomato rhizosphere bacterium *Pseudomonas* spp., it is imperative to delve into the investigation of siderophore-mediated effects utilizing standard siderophores.

### Effects mediated by other metabolites on pathogen invasion under iron-limited condition

Apart from siderophores, many secondary metabolites can directly inhibit the growth of soil-borne pathogens. For instance, hydrogen cyanide produced by fluorescent pseudomonads showed toxicity against phytopathogens [57], while pyrrolnitrin was reported to possess antagonistic activity against fungi, yeast, and gram-positive bacteria, which involves inhibition of glycerol kinase that leads to leakage in the cell membrane via accumulation of glycerol by inhibiting cytochrome c oxidase [58]. Our findings indicate that other metabolites produced by *Pseudomonas* strains suppressed the growth of the tomato pathogen *R. solanacearum* QL-Rs1115. However, as environmental conditions shift from iron-rich to iron-limited, the inhibitory effect of these metabolites on pathogenic bacteria diminishes. This attenuation of inhibitory effect coincides with a decrease in PG content, yet without significant differences in DAPG gene expression observed, which indicates that the regulation of PG production by iron may be dependent on post-transcriptional mechanisms [40]. This suggests that the reduction in inhibition may be attributable to growth limitations imposed by iron scarcity. Essentially, the diminished antagonistic effect of these metabolites on the tomato pathogen could stem from the constraints on growth antagonism induced by limited iron availability.

Iron deficiency dramatically affects the utilization of metabolic pathways related to iron, favoring iron-independent pathways while curtailing iron-dependent ones [59]. This alteration prompts a microbial adaptation through the regulatory adjustments of secondary metabolites to thrive in iron-constrained environments. Given that these environmental changes often occur concurrently, microbial cells likely adjust their metabolism to respond effectively. For instance, under sulfur limitation, *E. coli* experiences reduced sulfide and adenosine 5′-phosphosulphate levels while increasing the N-acetylserine pool [60, 61]. Furthermore, microorganisms strategically balance investments in different metabolites to optimize environmental adaptation, exemplified by the investment balance between the antibiotics 2,4-DAPG and pyoluteorin from *Pseudomonas protegens* FD6 [62]. Analyses via HPLC revealed that the *ΔphlC* mutant lacked 2,4-DAPG biosynthesis, yet pyoluteorin production in this mutant significantly increased by 67-fold compared with the wild-type FD6 [62]. Similarly, the *ΔpltD* mutant exhibited a substantial decrease, >16-fold, in 2,4-DAPG levels compared with strain FD6 [62]. The iron-deficient environment not only stimulates siderophore production but may also influence the expression of other secondary metabolites. Our research indicates a correlation between the effects of other metabolites in tomato bacterial consortia and their siderophore production; consortia with high siderophore levels exhibited weakened inhibition mediated by other metabolites, whereas those with lower levels showed an increase in such inhibition of tomato soil-borne pathogen. This observation suggests a possible trade-off between the production of siderophores and other metabolites. Nonetheless, further detailed data are required to robustly support this hypothesis.

In this study, only seven *Pseudomonas* strains were used to construct microbial consortia. In reality, the plant-associated microbiome is considerably more intricate, with interactions mediated by siderophores within the comprehensive microbiome remaining largely unexplored. This study reveals the non-negligible roles of siderophores in mediating bacterial interactions and competing iron with the soil-borne pathogen *R. solanacearum* under iron-limited rhizosphere environments, which is in line with our previous studies [13]. However, bacterial siderophores exhibit diverse structures, even within a single strain [24], and numerous siderophores remain challenging to isolate and purify using current technologies [63]. This study aimed to comprehensively assess the effects of all siderophores by comparing bacterial interactions under iron-limited and iron-rich conditions. Furthermore, it remains uncertain whether the siderophore absorption systems of different phylogenetic *Ralstonia* strains are comparable. Exploring different phylotypes of pathogenic *Ralstonia* strains within varying soil–host systems could provide valuable insights. Plant-associated microbes produce an array of secondary metabolites. While this investigation primarily focused on comparing the importance of siderophores and 2,4-DAPG produced by *Pseudomonas* strains under iron-limiting conditions, future studies could delve into the significance of siderophores and other secondary metabolites (such as iturin, macrolactin) produced by indigenous microbes in similar iron-limited scenarios.

## Conclusions

We conclude that siderophores play a pivotal role in the inhibition of plant-pathogenic bacteria by root-associated microorganisms. Under the iron-limited conditions prevailing on tomato roots, the effect of other metabolites was diminished, while increased siderophore production led to heightened inhibitory effects of root-dwelling bacterial strains. Our findings suggest that within the bacterial consortia comprising *Pseudomonas* strains, siderophore-mediated interactions offer a valuable predictive indicator of their ability to suppress the tomato pathogenic bacterium *R. solanacearum*. Given the widespread prevalence of iron deficiency in soil environments, siderophores assume a significant role in shaping interactions between beneficial and pathogenic bacteria. It is essential to consider not only the direct inhibitory effects of siderophores but also their mediated interactions among beneficial bacteria, particularly within the intricate plant rhizosphere. While bacteria release a wide array of metabolites, the importance of siderophore-mediated effects should not be underestimated, despite the critical roles played by other secondary metabolites in bacterial interactions of tomato plants. Moreover, the nutritional status of the environment should be a key consideration when implementing biological control strategies for tomato diseases.

## Materials and methods

### Bacterial strains and construction of inoculated consortia

In our study, we used seven fluorescent *Pseudomonas* strains, specifically CHA0, Q2-87, Q8R1-96, 1 M1-96, MVP1-4, F113, and Phl1C2, as previously described [64]. These strains were kept at −80°C for long-term storage. Before conducting the experiments, a single colony of each strain was randomly selected. These colonies were cultured overnight in lysogenic broth (LB), followed by a series of three washes using 0.85% NaCl solution. Subsequently, the cultures were adjusted to an optical density at 600 nm (OD_600_) of 0.5 using a spectrophotometer (Spectra Max M5, Molecular Devices, Sunnyvale, CA, USA). We used a modified King's B medium (MKB: 2.5 g l^−1^ K_2_HPO_4_, 2.5 g l^−1^ MgSO_4_·7H_2_O, 15 ml l^−1^ glycerin, and 5.0 g l^−1^ casamino acids, pH 7.2, iron concentration ~6 μM) as the iron-limited environment to induce the production of siderophores [37]. To obtain iron-rich conditions (Fe(III) concentration = 50 μM) we supplemented the medium with 50 μM FeCl_3_, thereby eliminating the impact of iron limitation and siderophore-mediated interactions. Conversely, the iron concentration in the iron-limited MKB medium was ~6 μM [37, 65], allowing bacterial growth to some extent, regardless of their ability to produce siderophores. The pathogen employed in our investigation was *R. solanacearum* strain QL-Rs1115, known for its high virulence and capability to induce wilting in tomato plants. The strain was originally isolated from tomato rhizosphere in Qilin (118°57′ E, 32°03′ N), Nanjing, China [66]. All bacterial strains are listed in Supplementary Data Table S1.

By the use of seven *Pseudomonas* strains, a total of 49 consortia were created, covering a richness gradient of one, two, four, and six strains (one strain, 7 treatments; two strains, 21 treatments; four strains, 18 treatments; six strains, 3 treatments; Supplementary Data Table S2). Each consortium was standardized to a final bacterial density of 10^7^ cells ml^−1^, with equal representation of all strains involved. The inoculum volume of each bacterial community was consistent, while the proportion of individual strains was adjusted inversely with increasing community richness (100, 50, 25, and 16.7% for consortia of one, two, four, and six strains, respectively). The same experimental design was followed in both the *in vitro* and greenhouse experiments.

### Determining the siderophore production of consortium members and bacterial consortia

Siderophore production in bacterial strains and consortia was measured by chrome azurol S (CAS) assays, which gauge the intensity of siderophores chelating ferric ions based on the resulting color change in the reaction mixture [67]. All *Pseudomonas* strains and bacterial consortia were cultured (30°C, 48 h, 170 r.p.m.) in MKB medium and iron-rich MKB medium (MKB + Fe), respectively. Cell-free supernatants were obtained by centrifugation (6000 r.p.m. for 10 min) and subsequent filtration using a 0.22-μm filter and then assayed for siderophore production by a modified version of the universal CAS assay developed by Schwyn and Neilands [67]. Briefly, 100 μl of each cell-free supernatant (three biological replicates for all 49 bacterial consortia) or fresh medium as a control, was combined with 100 μl of the CAS assay solution (containing 1.5 × 10^−3^ mM FeCl_3_·6H_2_O, 6 × 10^−1^ mM adecyltrimethylammonium bromide, 4.307 g anhydrous piperazine, and 1.5× 10^−1^ mM CAS) in a 96-well microplate. Following 1 h of static cultivation at room temperature, the optical density at 630 nm (OD_630_) of cell-free supernatant (A) and the uninoculated medium control (Ar) were measured using a spectrophotometer (SpectraMax M5, Sunnyvale, CA, USA). Deferoxamine B siderophore was utilized as the standard to create standard curves for assessing siderophore production in both *Pseudomonas* strains and microbial communities. Employing the observable color change of the CAS solution upon siderophore presence, diverse dilutions of deferoxamine B siderophore, in combination with OD_630_ measurements, were pivotal in constructing these standard curves. Siderophore concentration was normalized as deferoxamine (DFO) equivalent and expressed as log_10_DFO. To be noted, the siderophore concentration of the supernatant collected under iron-limited conditions required dilution with sterile water due to saturation in the assay.

### Determining the phloroglucinol production and antibiotic gene expression of consortium members under different iron conditions

Phloroglucinol (PG), an intermediate product of DAPG [68, 69], was quantified in the supernatant using a method modified from a previous study [70]. Seven *Pseudomonas* strains were cultured in iron-rich and iron-limited media for 48 h, with each treatment carried out in triplicate. Cell-free supernatants were collected as mentioned above. To each sample (75 μl of supernatant), 25 μl of HCl was added. Following this, 100 μl of cinnamaldehyde–HCl reagent, comprising 0.2% 4-hydroxy-3-methoxy-cinnamaldehyde in HCl:ethanol (1:3, v/v), was introduced to the solution, resulting in a pink coloration in samples containing PG. The colorimetric reaction was allowed to proceed for 2 h, after which the absorbance was measured at 550 nm. The absolute concentration of PG was subsequently determined by referencing a standard curve constructed from varying concentrations of PG standards.

To determine the effect of iron deficiency on antibiotic gene expression, the expression of the *phlD* gene was assessed under different iron conditions by RT–qPCR. Seven *Pseudomonas* strains were cultured in iron-rich and iron-limited MKB media for 48 h. The total RNA of the bacteria was extracted according to the protocol of the Bacterial RNA Kit (R6950, Omega, USA). The concentration and purity of RNA were determined using a NanoDrop 1000 spectrophotometer (Thermo Scientific, Waltham, MA, USA), with A260/A280 and A260/230 ratio values of around 2 considered adequate for inclusion in the study. cDNA was synthesized with the HiScript^®^ II Q RT SuperMix for qPCR (+gDNA wiper) Kit (Vazyme, China). For RT–qPCR analysis, two primers specific to the *phlD* gene were used: B2BF (5′-ACC CAC CGC AGC ATC GTT TAT GAGC-3′) and B2BR3 (5′-AGC AGA GCG ACG AGA ACT CCA GGG A-3′) [71]. The RT–qPCR assays were performed in the Applied Biosystems 7500 Real-Time PCR System (Applied Biosystems, CA, USA) using ChamQ SYBR Color qPCR Master Mix (Vazyme, China). Each reaction mix, with the total volume of 20 μl, included 10 μl of 2 × ChamQ SYBR Color qPCR Master Mix (High ROX Premixed), 1 μl of cDNA, 0.4 μl of both forward and reverse primers, and 8.2 μl of ddH_2_O. The PCR was performed by initially denaturing at 95°C for 30 s and cycling 40 times at 95°C for 10 s, 60°C for 30 s, and melt curve analysis at 95°C for 15 s, 60°C for 1 min, and 95°C for 15 s. Each quantification was conducted in duplicate. Gene expression analysis was performed using the 2^−△△Ct^ method [72]; the expression of *phlD* genes was given as mean fold-change in gene expression normalized to a reference gene (*recA*) [73].

### Determining the pathogen resistance of inoculated consortia using a co-culture experiment

The invading pathogen used in this study was the *R. solanacearum* strain QL-Rs1115 (GenBank accession GU390462), which was tagged with the pYC12-mCherry plasmid [14]. To assess the influence of siderophores produced by individual consortium members and bacterial consortia on pathogen growth, we conducted co-culture experiments by combining each consortium member or bacterial consortium with *R. solanacearum* in both iron-rich MKB medium (MKB + Fe) and iron-limited medium (MKB) (Fig. 1C). Each consortium member or bacterial consortium was co-cultured with an equal volume of the pathogen *R. solanacearum*, both at an OD_600_ of 0.5. Specifically, 2 μl of each culture was combined in the 96-well plates containing 196 μl iron-limited MKB medium at 30°C with shaking (170 r.p.m.). As a control, 2 μl of sterile distilled water was used in place of the inoculated consortium. All co-culture experiments were carried out in triplicate. Optical density (600 nm) was recorded at regular intervals with a spectrophotometer, while pathogen density was determined based on the mCherry fluorescence signal (excitation, 587 nm; emission, 610 nm). Pathogen abundance during co-culture with *Pseudomonas* consortia was indicated by pathogen density relative to total bacterial density [log_10_ (RFP/OD_600_)]. By comparing the pathogen abundance of the inoculated consortia to the control treatment, the resistance of the consortia against pathogenic bacteria was quantified. The efficacy of resisting invasion was indicated by pathogen abundance being lower than that in the control, with decreased pathogen abundance correlating to enhanced resistance. A consortium displaying strong resistance will exhibit the lowest pathogen abundance in the co-culture setting. The ability of the consortium to suppress pathogen invasion was quantified using the following formula: inhibition rate (%) = (1 − RFP_c_/RFP_rs_) × 100%, where RFP_rs_ denotes the density of the pathogen [log_10_ (RFP/OD_600_)] in monoculture, while RFP_c_ denotes the density of the pathogen when the consortium was co-cultured with the pathogen. Consortia exhibiting higher inhibition rates possess a stronger capacity to suppress pathogen invasion.

### Determining siderophore-mediated effects and other metabolite-mediated effects using the supernatant experiment

To explore the metabolite-mediated effect of inoculated consortia or consortium members on strain growth, cell-free supernatants from 49 consortia were collected as described above (Fig. 1A and B). The following three treatments were set up to distinguish siderophore-mediated effects from other metabolite-mediated effects [33]. (i) Iron-limited: 20 μl of cell-free supernatant collected under iron-limited conditions was added to 178 μl of MKB medium. This supernatant contained total metabolites produced by consortia or consortium members in iron-limited environments, including siderophores (SM). (ii) Iron-replenish: 20 μl of cell-free supernatant collected under iron-limited conditions was added to 178 μl of iron-rich MKB medium. This supernatant was supplemented with 50 μM FeCl_3_ to chelate siderophores via an iron-chelation reaction (M). (iii) Iron-rich: 20 μl of cell-free supernatant collected under the iron-rich condition was added to 178 μl of iron-rich MKB medium. This supernatant contained total metabolites under iron-rich conditions, excluding siderophores produced (MC). Following this, each treatment was inoculated with 2 μl of QL-Rs1115 (adjusted to OD_600_ = 0.5 after 12 h of growth at 30°C with shaking) and inoculated for 24 h with agitation (170 r.p.m.) at 30°C. Optical density (600 nm) was recorded at regular intervals using a spectrophotometer. The effect of each supernatant on pathogen growth (SNG) was calculated as the relative growth effect (RG) compared with the water control (CG), where sterile water was used instead of the supernatant. The formula used for calculation was: RG = [(SNG ÷ CG) – 1] × 100, where SNG refers to SM, M, and MC supernatants as described above. RG values below or above zero thus indicated growth inhibition and facilitation, respectively. By adding enough FeCl_3_ in the iron-replenish treatment, the siderophores in the supernatant were inactivated, enabling the assessment of effects mediated by other metabolites (excluding siderophores) on pathogen growth (Fig. 1A). Subsequently, the manifestation of siderophore-mediated effects was accomplished by contrasting the overall metabolite-mediated impact with the effects specifically mediated by other metabolites under iron-limited conditions (SM − M = S) [33]. All supernatant assays were carried out in triplicate.

To calculate the mean siderophore and other metabolite-mediated growth effects by multi-strain consortia on each consortium member, different supernatants (SM, M, and MC) from all bacterial consortia were collected as mentioned above and then inoculated with seven *Pseudomonas* strains. The effect of different supernatants on the growth of consortium members was calculated as the relative growth effect compared with the water control treatment, using the previously described method. The interaction within bacterial consortia was represented by calculating the average effect of consortium metabolites on each consortium member. The calculation of the interactions within bacterial consortia was carried out according to the formula [e.g. interaction within consortium*_ij_* = $\frac{1}{n}\sum (\mathrm{RG}i+\mathrm{RG}j$)], where consortium*_ij_* comprises two species *i* and *j*, RG*i* represents the supernatant effects of consortium*_ij_* on species *i,* RG*i* represents supernatant effects on species *j*, *n* represents the number of species in the community (which is 2 for consortium*_ij_*) sourced from a prior study [33]. Siderophore-mediated interactions within consortia were calculated from 42 consortia, excluding single-species consortia.

### Determining disease resistance of tomato plants inoculated with *Pseudomonas* consortia via greenhouse experiment

To evaluate the ability of *Pseudomonas* consortia to suppress pathogen invasion in the natural plant rhizosphere, pot experiments were performed in a greenhouse using tomato seedlings (Fig. 1D). Surface-sterilized tomato seeds (*Lycopersicon esculentum*, cultivar ‘Jiangshu’) were germinated on water-agar plates for 3 days before being sown on seedling plates containing seedling substrate (Huainong, Huai’an Soil and Fertilizer Institute, Huai’an, China). Following germination, tomato seedlings at the three-leaf stage were transplanted into six-well trays, each well containing 100 g of seedling substrate. Seven days post-transplantation, a total of 49 bacterial consortia were inoculated to the roots of the seedlings using a drenching method (final concentration of 10^7^ cells g^−1^ soil). Each treatment involved three technical replicates, with one replicate comprising a six-well tray holding six tomato seedlings (totaling 18 seedlings per treatment). As a control measure, sterile water was used instead of the bacterial consortium. One week after inoculation with bacterial consortia, the invasion experiment proceeded with inoculation with the pathogen *R. solanacearum* (final concentration of 10^6^ cells g^−1^ soil). After inoculation with bacterial consortia, the plants were grown in a glasshouse for 49 days under natural temperature conditions ranging from 25 to 35°C and humidity levels between 60 and 80%. The plants were watered daily with sterile water, and the seedling trays were randomly rearranged every 3 days. The invasion experiments continued for 42 days after inoculation with the pathogen *R. solanacearum*, during which the disease incidence (DI) of each tomato seedling was recorded daily after the first disease symptoms appeared. DI was evaluated based on a disease index (di) scale ranging from 0 to 4, where 0 = no wilting; 1 = 1–25% wilting; 2 = 26–50% wilting; 3 = 51–75% wilting; and 4 = 76–100% wilted or dead. DI = [∑(number of diseased plants in this index × di)/(total number of plants investigated × highest di)] × 100% [66]. The area under the disease progress curve (AUDPC) was calculated using the disease incidence data, a method commonly used to consolidate multiple observations of disease progress into a single value for analysis [74].

### Quantification and statistical analyses

Student's *t*-tests were performed to evaluate statistical significance for pathogen inhibition rate, effects mediated by other metabolites, PG content, expression of antagonistic genes, biomass, and siderophore yield between iron-rich and iron-limited conditions. Wilcoxon rank-sum tests were utilized to assess the statistical significance of differences among different metabolite-mediated effects of consortia. *P* values below 0.05 were deemed statistically significant. Treatment of the experimental results was based on analyzing averages and ANOVA. When the ANOVA indicated a significant difference among factors, the Duncan test was used to identify groups that were significantly different from one another. To analyze the effects of metabolites on the inhibition rate (co-culture) and disease incidence (greenhouse experiment), AUDPC (greenhouse experiment) and the effect of siderophore on other metabolite-mediated effects, generalized linear models were used. All variables were fitted as continuous variables and one separate model was used for each variable. Finally, structural equation modeling (SEM) was used to elucidate the mechanisms underlying disease incidence in tomato plants, considering multiple potentially correlated effect pathways. SEM (lavaan package in R) was used to investigate the relative importance of siderophore production, siderophore-mediated effects, siderophore-mediated interactions, pathogen abundance, and AUDPC. The adequacy of the models was determined via χ^2^ tests, the Akaike information criterion (AIC), and root mean square error of approximation (RMSEA) [75]. Model modification indices and stepwise removal of non-significant relationships were used to improve the models; however, only scientifically sound relationships were considered. All data analyses were performed using R version 4.1.0.

## Supplementary Material

Web_Material_uhae186

## Data Availability

The data for this article are available in the article or in its supplementary material.
