# Supplementary material for: Siderophore interactions drive the ability of Pseudomonas spp. consortia to protect tomato against Ralstonia solanacearum
Source: Hortic Res. 2024 Jul 12;11(9):uhae186. doi: 10.1093/hr/uhae186 (PMC11377186; doi:10.1093/hr/uhae186)
Supplement: Web_Material_uhae186 [file web_material_uhae186.zip › Supplementary_0621.docx]

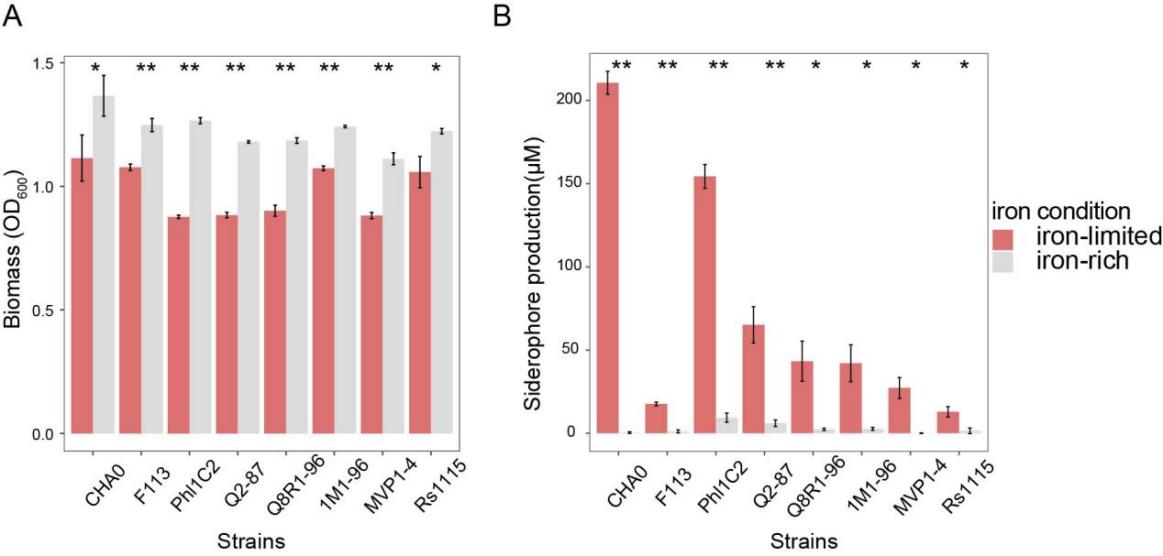


**Figure S1. Biomass and siderophore production of *Pseudomonas* strains under different iron conditions.** Biomass (A) and siderophore production (measured with the CAS assay) of *Pseudomonas* strains and pathogen *Ralstonia solanacearum* strain QL-Rs1115 under iron-limited and iron-rich conditions (B). “ns” and “*” indicate absence and presence of significant differences (Student’s t-test, *: *P* < 0.05, **: *P* < 0.01) between different iron treatments, respectively.


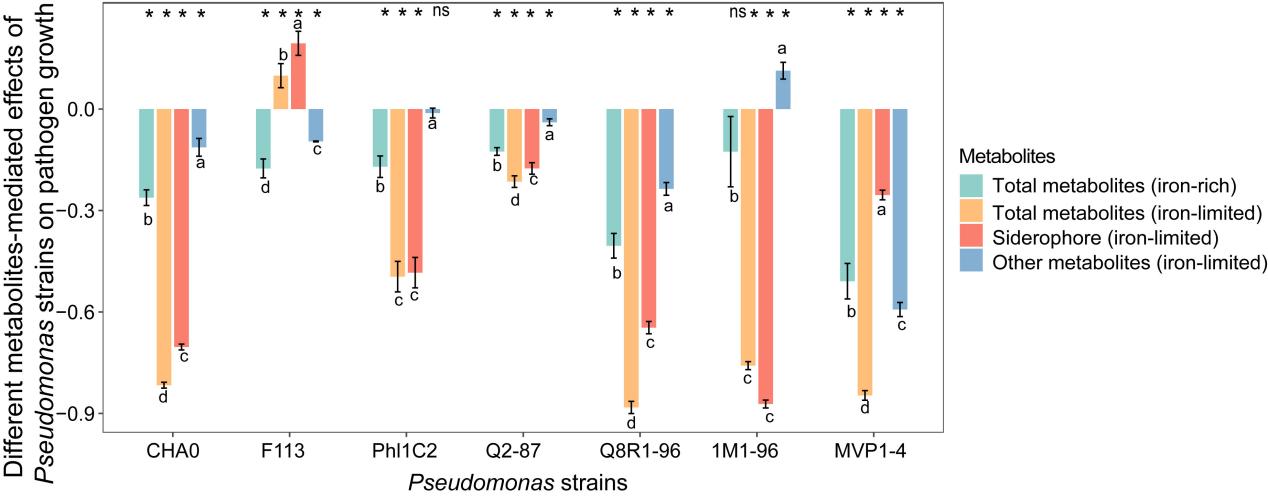


**Figure S2. Metabolite-mediated effects of *Pseudomonas* strains on *Ralstonia solanacearum* strain QL-Rs1115 growth in supernatant experiments.** Different colors represent different metabolite-mediated effects on pathogen growth, with each color corresponding to cell-free supernatants cultured under different iron conditions. “Total metabolites (iron-rich)” and “Total metabolites (Iron-limited)” represents total metabolites collected in iron-rich and iron-limited conditions, respectively. Siderophore-mediated effects was determined by subtracting other metabolite-mediated effects from the total metabolites. “Other metabolites” represent remaining metabolites by excluding siderophores from those collected in iron-limited conditions, achieved by adding enough FeCl_3_ to the iron-limited supernatant. Different letters indicate significant differences between the groups (*P* < 0.05) using ANOVA and Duncan tests. “*” indicates the presence of significance (Student’s t-test, *: *P* < 0.05, **: *P* < 0.01) in the effects of supernatant on pathogen growth. It was defined that siderophore-mediated effects between 0.15 and -0.15 were considered non-significant differences.


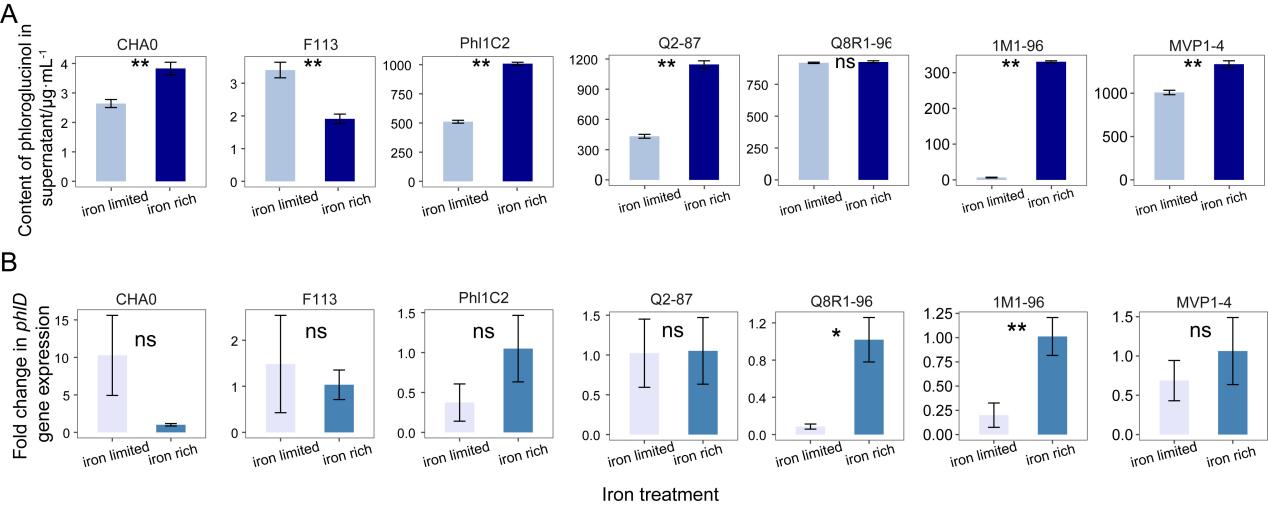


**Figure S3. The effects of iron restriction on the production and gene expression of antagonistic substances.** (A) The content of phloroglucinol in the supernatant of *Pseudomonas* strains under different iron conditions. (B) *phlD* gene expression of *Pseudomonas* strains under different iron conditions. “ns” and “*” indicate absence and presence of significant differences (Student’s t-test, *: *P* < 0.05, **: *P* < 0.01) between different iron treatments.


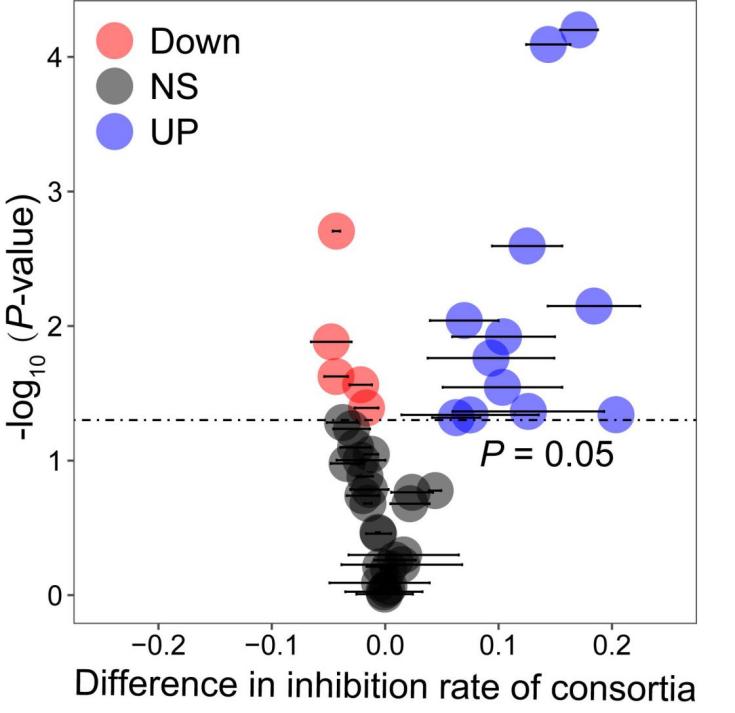


**Figure S4. Change in pathogen inhibition rate of *Pseudomonas* consortia.** Each point represents the variation in inhibition rate of each *Pseudomonas* consortium when co-cultured with the pathogen. The Y-axis represents the significance of the difference, while the X-axis represents the variance of inhibition rate between iron-limited and iron-rich conditions. The dashed line represents *P* = 0.05. Different colors represent distinct alterations in the inhibition rate. Blue represents an increase in the inhibition rate of consortia under iron-limited conditions compare to iron-rich conditions, red represents a decrease, and black represents no significant difference (NS, *P* > 0.05).

**
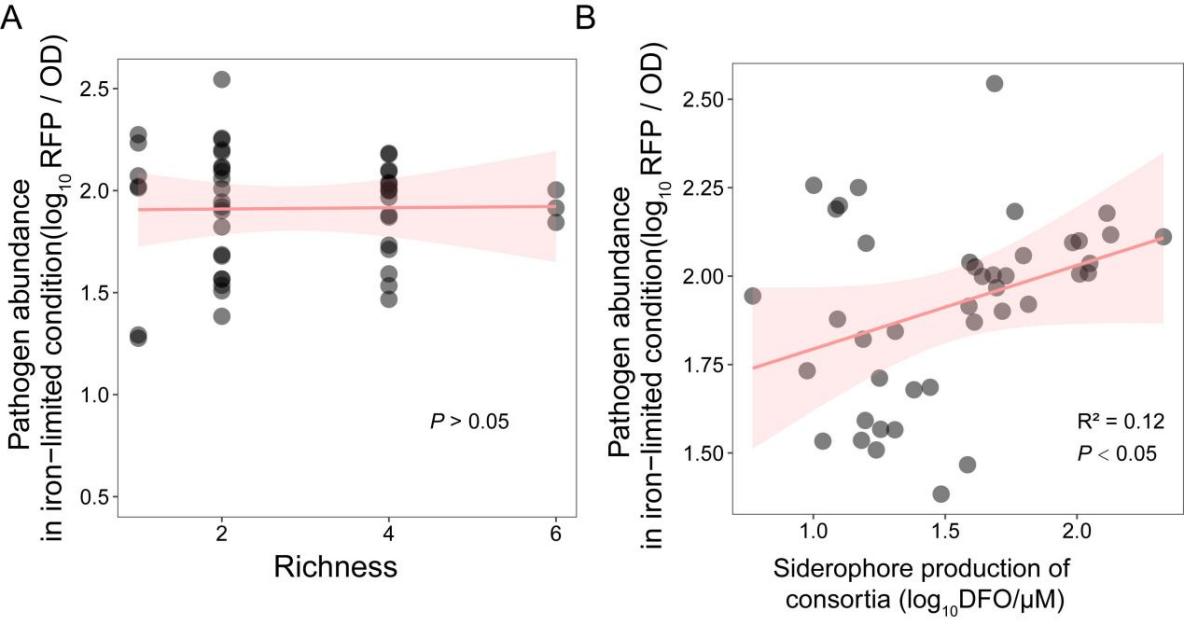
**

**Figure S5. Siderophore production and richness of consortia and their ability to suppress pathogen invasion.** (A) There was no significant correlation between pathogen abundance when co-culture with bacterial consortium and the richness of consortia. (B) Co-culture experiments with the *Ralstonia solanacearum* pathogen and individual bacterial consortium revealed a correlation between pathogen abundance and siderophore production under iron-limited conditions (R^2^ = 0.12, F_1,40_ = 5.54, *P* < 0.05).

**
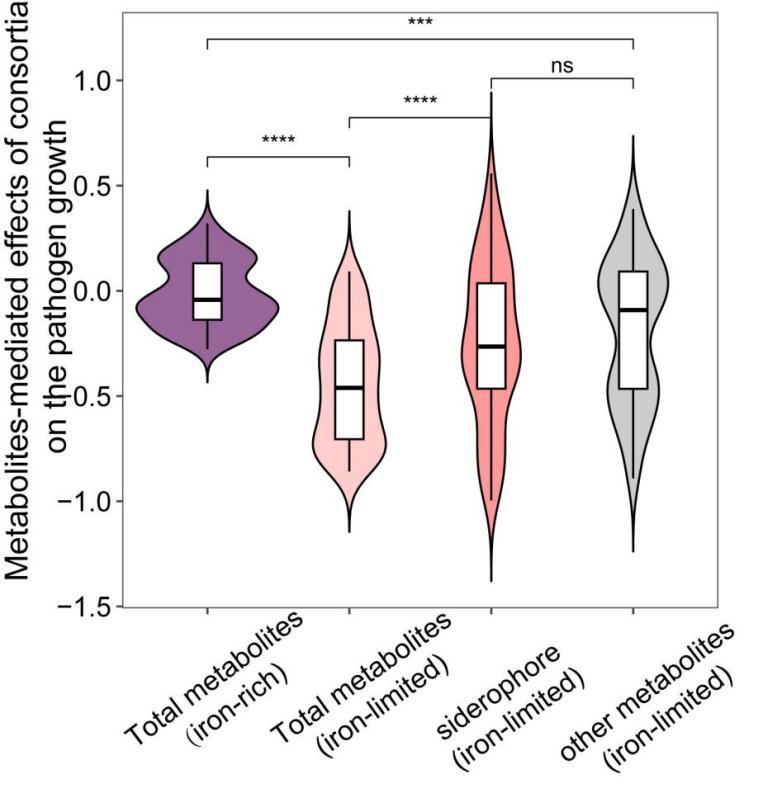
**

**Figure S6. Metabolite-mediated effects of *Pseudomonas* consortia on *Ralstonia solanacearum* QL-Rs1115 growth under different iron conditions.** The effects of different secondary metabolites on the pathogen growth under different iron conditions. “Total metabolites (iron-rich)” and “Total metabolites (Iron-limited)” represents total metabolites collected in iron-rich and iron-limited conditions. Siderophore-mediated effects was determined by subtracting other metabolite-mediated effects from the total metabolites. “Other metabolites” represent remaining metabolites by excluding siderophores from those collected in iron-limited conditions, achieved by adding enough FeCl_3_ to the iron-limited supernatant. “ns” and “*” indicate absence and presence of significant differences (Wilcoxon test, *P* < 0.05) between different metabolites.


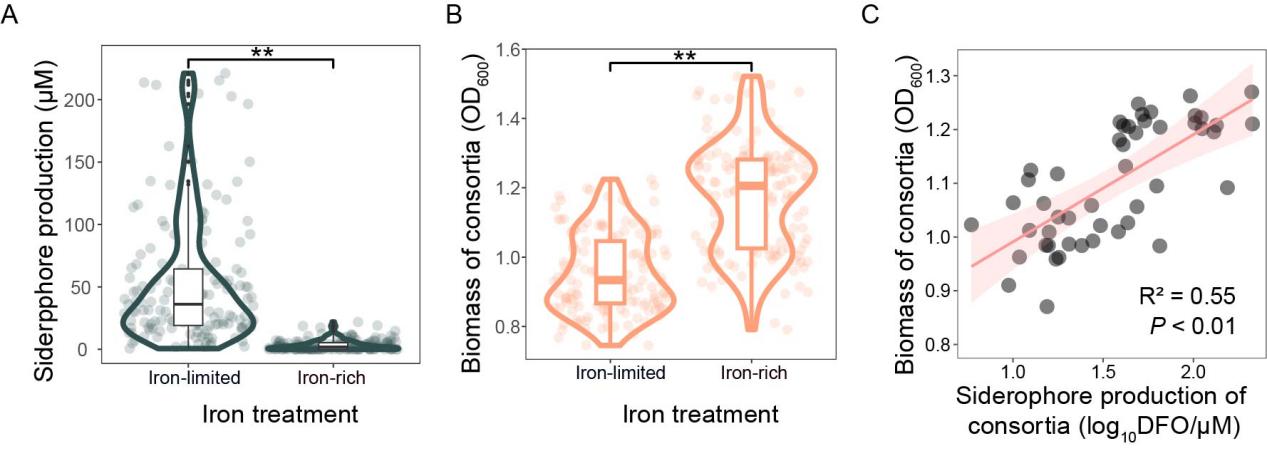


**Figure S7. Siderophore production and biomass of *Pseudomonas* consortia under different iron conditions and their correlation analysis.** (A) Siderophore production of *Pseudomonas* consortia under different iron conditions. (B) Biomass of *Pseudomonas* consortia under different iron conditions. (C) Correlation between biomass and siderophore production by the inoculated *Pseudomonas* consortia under iron-limited conditions (R^2^ = 0.55, F_1,47_ = 57.37, *P* < 0.01). “ns” and “*” indicate absence and presence of significant differences (Student’s t-test, *: *P* < 0.05, **: *P* < 0.01) between different iron treatments.

**
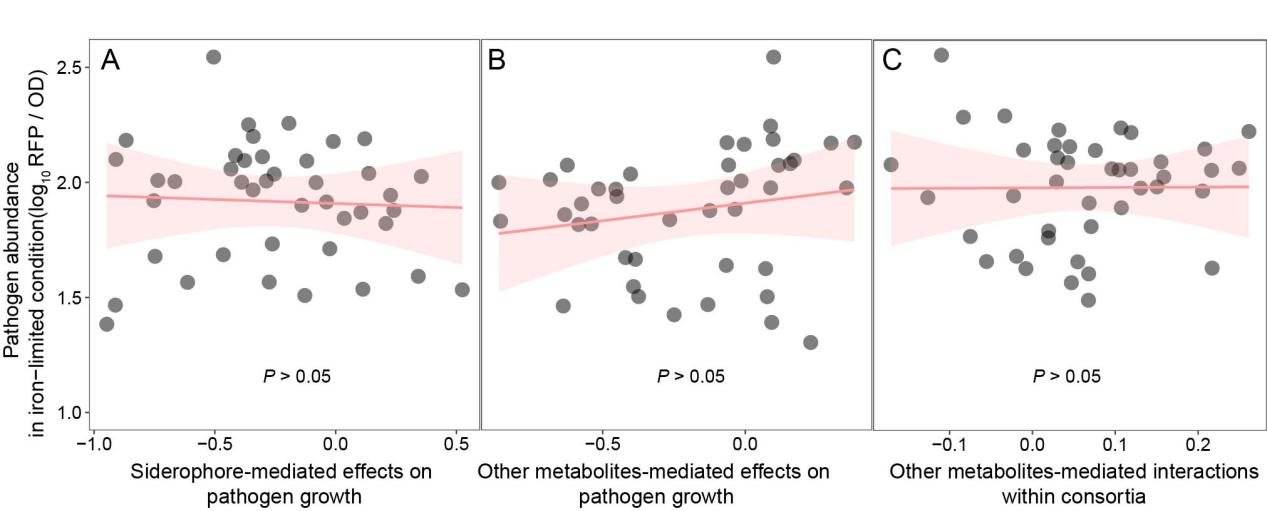
**

**Figure S8. Differential metabolite-mediated effects on *Ralstonia solanacearum* QL-Rs1115 growth and other metabolites-mediated interactions within consortia under iron-limited conditions and pathogen abundance in the coculture experiment.** There was no correlation between pathogen abundance and the metabolite-mediated effects—either siderophore (A) or other metabolites (B). There was no correlation between pathogen abundance and the other metabolites-mediated interactions within consortia (C).

**
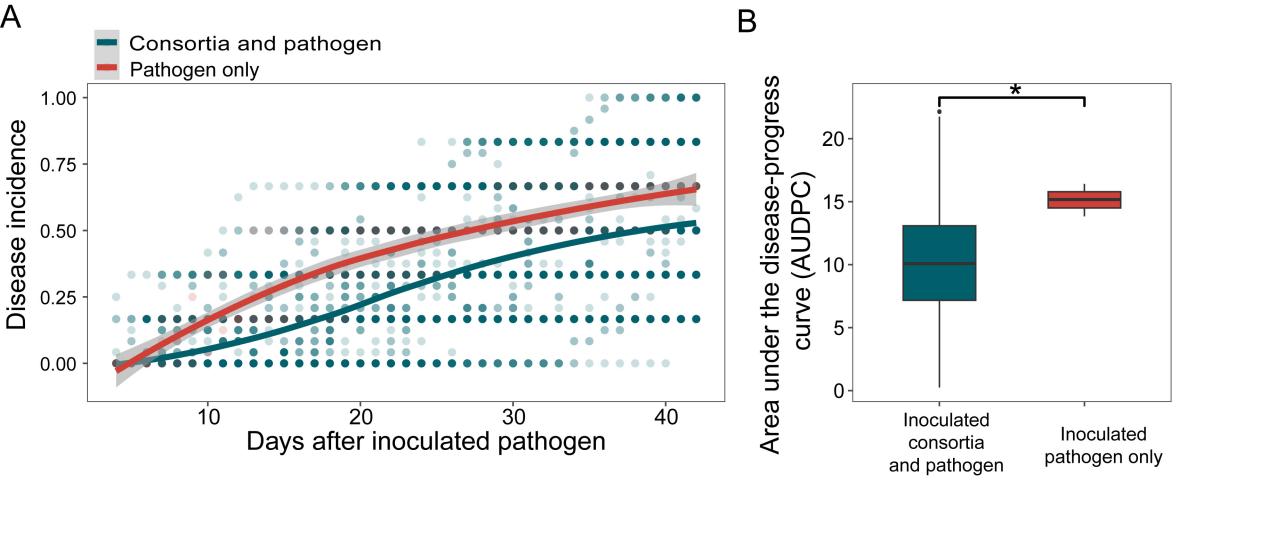
**

**Figure S9. Progression and severity index of bacterial wilt plant disease in the absence and presence of inoculated consortia.** (A) Disease spread was fitted with the data using a logistic regression to obtain three variables describing the dynamics of disease. Different colors represent tomato plants inoculated with *Pseudomonas* consortia and those without, while red indicating plants inoculated with the pathogen alone. (B) 42 days after inoculated with pathogen, disease severity index (AUDPC) of tomato seedlings inoculated with the consortia significantly differed from those inoculated with the pathogen alone (t = -6.16, df = 2.54, *P* < 0.05).

**
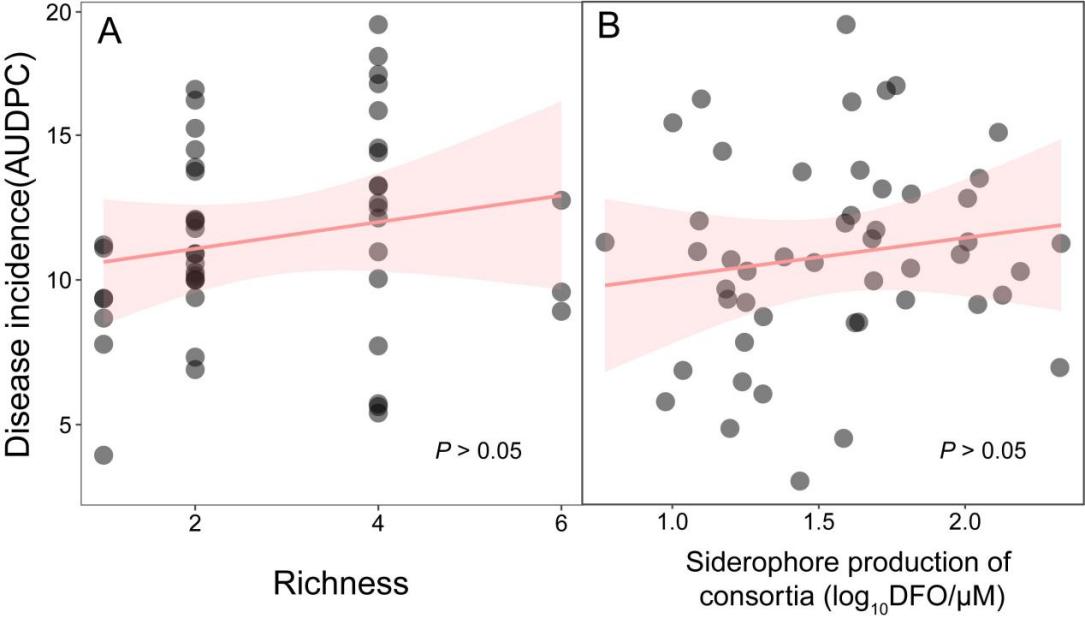
**

**Figure S10. Richness and siderophore production of consortia and their ability to suppress pathogen invasion in greenhouse experiments.** (A) There was no significant correlation between the richness of consortia and disease incidence(AUDPC) of plants inoculated with the consortia. (B) There was no significant correlation between the siderophore production of consortia and and disease incidence (AUDPC) of plants inoculated with the consortia.


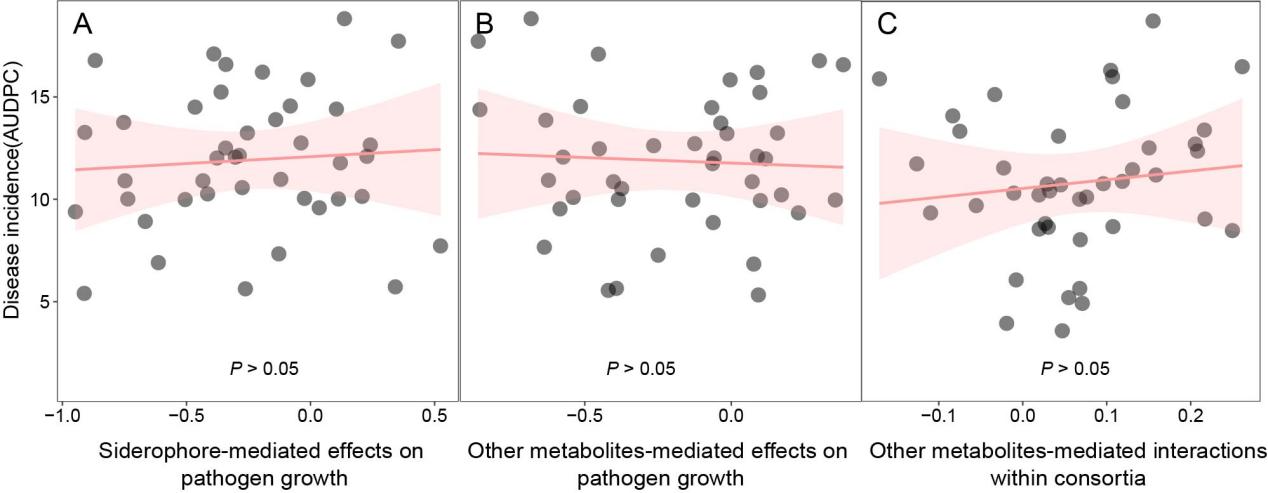


**Figure S11. Different metabolite-mediated effects on pathogen growth and other metabolites-mediated interactions within consortia and their ability to suppress pathogen invasion.** There was no significant linear correlation between the disease incidence (quantified as the area under disease progress curve, AUDPC) and metabolite-mediated effects—either siderophore (A) or other metabolites (B). Additionally, interactions between other metabolites-mediated interactions within consortia in the supernatant and the disease incidence (AUDPC) in greenhouse experiment were examined (C).


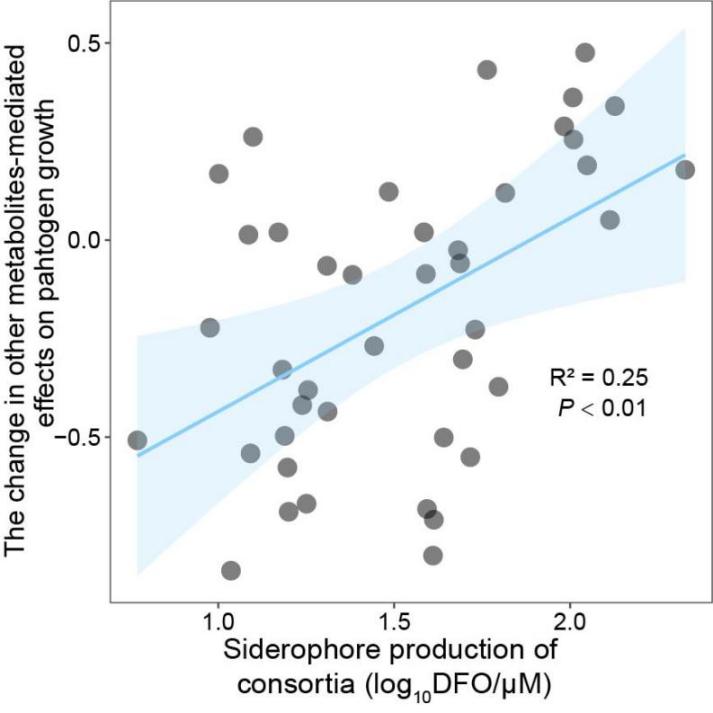


**Figure S12.** **The relationship between the changes in other metabolites-mediated effects on pathogen growth and siderophore production under iron-limited conditions.** The change value of other metabolites-mediated effects on pathogen growth in the supernatant was calculated by subtracting the effect under iron-limited conditions from the effect under iron-rich conditions.

**Table S1. Information of *Pseudomonads* strains used in this study**

| Strains | Origin | Bacteria |
| --- | --- | --- |
| CHA0 | Tobacco, Switzerland | *Pseudomonas protegens* |
| F113 | Sugar beet, Irland | *Pseudomonas fluorescens* |
| Phl1C2 | Tomato, France | *Pseudomonas fluorescens* |
| Pf-5 | Wheat, USA | *Pseudomonas protegens* |
| Q2-87 | Wheat, USA | *Pseudomonas fluorescens* |
| Q8R1-96 | Wheat, USA | *Pseudomonas brassicacearum* |
| 1M1-96 | Wheat, USA | *Pseudomonas fluorescens* |
| MVP1-4 | Pea, USA | *Pseudomonas fluorescens* |
| QL-Rs1115 | Tomato, China | *Ralstonia solanacearum* |

**Table S2. Composition of the *Pseudomonas* bacterial communities used in this study.**

| Communities/ Pseudomonas strains | CHA0 | F113 | Phl1C2 | Q2-87 | Q8R1-96 | 1M1-96 | MVP1-4 | Richness |
| --- | --- | --- | --- | --- | --- | --- | --- | --- |
| 1 | 1 | 0 | 0 | 0 | 0 | 0 | 0 | 1 |
| 2 | 0 | 1 | 0 | 0 | 0 | 0 | 0 | 1 |
| 3 | 0 | 0 | 1 | 0 | 0 | 0 | 0 | 1 |
| 4 | 0 | 0 | 0 | 1 | 0 | 0 | 0 | 1 |
| 5 | 0 | 0 | 0 | 0 | 1 | 0 | 0 | 1 |
| 6 | 0 | 0 | 0 | 0 | 0 | 1 | 0 | 1 |
| 7 | 0 | 0 | 0 | 0 | 0 | 0 | 1 | 1 |
| 8 | 1 | 1 | 0 | 0 | 0 | 0 | 0 | 2 |
| 9 | 1 | 0 | 1 | 0 | 0 | 0 | 0 | 2 |
| 10 | 1 | 0 | 0 | 1 | 0 | 0 | 0 | 2 |
| 11 | 1 | 0 | 0 | 0 | 1 | 0 | 0 | 2 |
| 12 | 1 | 0 | 0 | 0 | 0 | 1 | 0 | 2 |
| 13 | 1 | 0 | 0 | 0 | 0 | 0 | 1 | 2 |
| 14 | 0 | 1 | 1 | 0 | 0 | 0 | 0 | 2 |
| 15 | 0 | 1 | 0 | 1 | 0 | 0 | 0 | 2 |
| 16 | 0 | 1 | 0 | 0 | 1 | 0 | 0 | 2 |
| 17 | 0 | 1 | 0 | 0 | 0 | 1 | 0 | 2 |
| 18 | 0 | 1 | 0 | 0 | 0 | 0 | 1 | 2 |
| 19 | 0 | 0 | 1 | 1 | 0 | 0 | 0 | 2 |
| 20 | 0 | 0 | 1 | 0 | 1 | 0 | 0 | 2 |
| 21 | 0 | 0 | 1 | 0 | 0 | 1 | 0 | 2 |
| 22 | 0 | 0 | 1 | 0 | 0 | 0 | 1 | 2 |
| 23 | 0 | 0 | 0 | 1 | 1 | 0 | 0 | 2 |
| 24 | 0 | 0 | 0 | 1 | 0 | 1 | 0 | 2 |
| 25 | 0 | 0 | 0 | 1 | 0 | 0 | 1 | 2 |
| 26 | 0 | 0 | 0 | 0 | 1 | 1 | 0 | 2 |
| 27 | 0 | 0 | 0 | 0 | 1 | 0 | 1 | 2 |
| 28 | 0 | 0 | 0 | 0 | 0 | 1 | 1 | 2 |
| 29 | 0 | 1 | 0 | 1 | 1 | 0 | 1 | 4 |
| 30 | 0 | 1 | 0 | 1 | 1 | 1 | 0 | 4 |
| 31 | 1 | 0 | 1 | 1 | 1 | 0 | 0 | 4 |
| 32 | 0 | 0 | 0 | 1 | 1 | 1 | 1 | 4 |
| 33 | 1 | 1 | 1 | 1 | 0 | 0 | 0 | 4 |
| 34 | 1 | 1 | 1 | 0 | 1 | 0 | 0 | 4 |
| 35 | 1 | 1 | 1 | 0 | 0 | 1 | 0 | 4 |
| 36 | 1 | 1 | 1 | 0 | 0 | 0 | 1 | 4 |
| 37 | 0 | 0 | 1 | 0 | 1 | 1 | 1 | 4 |
| 38 | 0 | 0 | 1 | 1 | 0 | 1 | 1 | 4 |
| 39 | 0 | 0 | 1 | 1 | 1 | 0 | 1 | 4 |
| 40 | 0 | 0 | 1 | 1 | 1 | 1 | 0 | 4 |
| 41 | 1 | 1 | 0 | 1 | 1 | 0 | 0 | 4 |
| 42 | 1 | 1 | 0 | 1 | 0 | 1 | 0 | 4 |
| 43 | 1 | 1 | 0 | 1 | 0 | 0 | 1 | 4 |
| 44 | 1 | 1 | 0 | 0 | 1 | 1 | 0 | 4 |
| 45 | 1 | 1 | 0 | 0 | 1 | 0 | 1 | 4 |
| 46 | 1 | 1 | 0 | 0 | 0 | 1 | 1 | 4 |
| 47 | 1 | 1 | 0 | 1 | 1 | 1 | 1 | 6 |
| 48 | 1 | 0 | 1 | 1 | 1 | 1 | 1 | 6 |
| 49 | 0 | 1 | 1 | 1 | 1 | 1 | 1 | 6 |

* **0 and 1 denote the absence and presence of *Pseudomonas* strains in a given community, respectively**

|  | **Siderophore mediated interaction within consortia** | | |
| --- | --- | --- | --- |
|  | df | F | P |
| Model 1-diversity effects |  |  |  |
| Strain richness |  |  |  |
| No. of Residuals |  | Not retained |  |
| Model summary |  | | |
| Model 2-identity effects |  |  |  |
| CHA0 | **↑1** | **12.54** | **<0.001** |
| F113 | **↑1** | **5.57** | **0.02** |
| Phl1C2 |  | Not retained |  |
| Q2-87 | **↓1** | **4.11** | **0.04** |
| Q8R1-96 |  | Not retained |  |
| 1M1-96 | **↓1** | **5.33** | **0.02** |
| MVP1-4 |  | Not retained |  |
| No. of Residuals | 139 |  |  |
| Model summary | R^2^ : 0.20 AIC : -97.13 | | |

**Table S3. ANOVA table on the effects of *Pseudomonas* community diversity and sampling effects on interactions within community. The significant effects (*P* < 0.05) are highlighted in bold and the ‘up’ and ‘down’ arrows denote for positive and negative effects, respectively.**

|  | **Siderophore production of consortia under iron-limited condition** | | |
| --- | --- | --- | --- |
|  | df | F | P |
| Model 1-diversity effects |  |  |  |
| Strain richness |  |  |  |
| No. of Residuals |  | Not retained |  |
| Model summary |  | | |
| Model 2-identity effects |  |  |  |
| CHA0 | **↑1** | **102.43** | **<0.001** |
| F113 | **↓1** | **16.04** | **<0.001** |
| Phl1C2 | **↓1** | **4.15** | **0.04** |
| Q2-87 |  | Not retained |  |
| Q8R1-96 | **↓1** | **37.80** | **<0.001** |
| 1M1-96 |  | Not retained |  |
| MVP1-4 | **↓1** | **11.50** | **0.05** |
| No. of Residuals | 139 |  |  |
| Model summary | R^2^:0.56 AIC: 1467.66 | | |

**Table S4. ANOVA table on the effects of probiotic *Pseudomonas* community diversity and sampling effects on siderophore production. The significant effects (*P* < 0.05) are highlighted in bold and the ‘up’ and ‘down’ arrows denote for positive and negative effects.**

**Table S5. Direct, total, and total indirect effects on disease incidence (AUDPC) of plant bacterial wilt and pathogen abundance for the structural equation model.**

| Variable | Total effects | | Direct effects | | Total indirect effects | |
| --- | --- | --- | --- | --- | --- | --- |
|  | Estimate | *P* | Estimate | *P* | Estimate | *P* |
| ***Effects on disease incidence*** |  |  |  |  |  |  |
| Interactions_sid | 0.372 | <0.001 | 0.225 | 0.008 | 0.147 | 0.001 |
| Biomass | 0.235 | 0.001 | NA | NA | 0.235 | 0.001 |
| Siderophore production | 0.214 | <0.001 | NA | NA | 0.214 | <0.001 |
| Pathogen abundance | 0.394 | <0.001 | 0.394 | <0.001 | NA | NA |
| ***Effects on pathogen abundance*** |  |  |  |  |  |  |
| Interactions_sid | 0.374 | <0.001 | 0.264 | 0.001 | 0.107 | 0.006 |
| Biomass | 0.596 | <0.001 | 0.596 | <0.001 | NA | NA |
| Siderophore production | 0.346 | <0.001 | -0.239 | 0.063 | 0.584 | <0.001 |

**Note: Interactions_sid indicates siderophore-mediated interactions within consortia in the supernatant experiments. NA denotes that the effect does not exist.**
